# Local Oxidation States in {FeNO}^6–8^ Porphyrins: Insights from DMRG/CASSCF–CASPT2 Calculations

**DOI:** 10.1021/acs.inorgchem.3c03689

**Published:** 2023-11-27

**Authors:** Quan Manh Phung, Ho Ngoc Nam, Abhik Ghosh

**Affiliations:** † Department of Chemistry, Graduate School of Science, Nagoya University, Furo-cho, Chikusa-ku, Nagoya, Aichi 464-8602, Japan; ‡ Institute of Transformative Bio-Molecules (WPI-ITbM), Nagoya University, Furo-cho, Chikusa-ku, Nagoya, Aichi 464-8601, Japan; § Institute of Materials Innovation, Institutes of Innovation for Future Society, Nagoya University, Furo-cho, Chikusa-ku, Nagoya, Aichi 464-8601, Japan; ∥ Department of Chemical Systems Engineering, Graduate School of Engineering, Nagoya University, Furo-cho, Chikusa-ku, Nagoya, Aichi 464-8603, Japan; ⊥ Department of Chemistry, UiT the Arctic University of Norway, N-9037 Tromsø, Norway

## Abstract

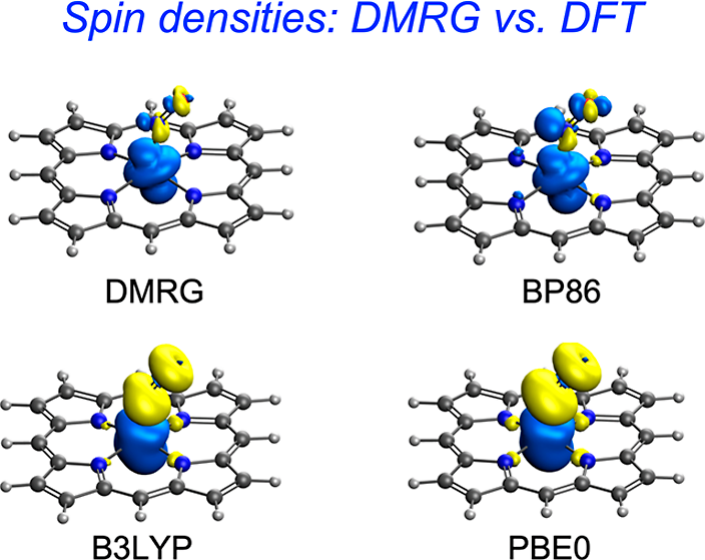

A first DMRG/CASSCF–CASPT2 study of a series of paradigmatic {FeNO}^6^, {FeNO}^7^, and {FeNO}^8^ heme–nitrosyl complexes has led to substantial new insight as well as uncovered key shortcomings of the DFT approach. By virtue of its balanced treatment of static and dynamic correlation, the calculations have provided some of the most authoritative information available to date on the energetics of low- versus high-spin states of different classes of heme–nitrosyl complexes. Thus, the calculations indicate low doublet–quartet gaps of 1–4 kcal/mol for {FeNO}^7^ complexes and high singlet–triplet gaps of ≳20 kcal/mol for both {FeNO}^6^ and {FeNO}^8^ complexes. In contrast, DFT calculations yield widely divergent spin state gaps as a function of the exchange–correlation functional. DMRG–CASSCF calculations also help calibrate DFT spin densities for {FeNO}^7^ complexes, pointing to those obtained from classic pure functionals as the most accurate. The general picture appears to be that nearly all the spin density of Fe[P](NO) is localized on the Fe, while the axial ligand imidazole (ImH) in Fe[P](NO)(ImH) pushes a part of the spin density onto the NO moiety. An analysis of the DMRG–CASSCF wave function in terms of localized orbitals and of the resulting configuration state functions in terms of resonance forms with varying NO(π*) occupancies has allowed us to address the longstanding question of local oxidation states in heme–nitrosyl complexes. The analysis indicates NO(neutral) resonance forms [i.e., Fe(II)–NO^0^ and Fe(III)–NO^0^] as the major contributors to both {FeNO}^6^ and {FeNO}^7^ complexes. This finding is at variance with the common formulation of {FeNO}^6^ hemes as Fe(II)–NO^+^ species but is consonant with an Fe L-edge XAS analysis by Solomon and co-workers. For the {FeNO}^8^ complex {Fe[P](NO)}^−^, our analysis suggests a resonance hybrid description: Fe(I)–NO^0^ ↔ Fe(II)–NO^–^, in agreement with earlier DFT studies. Vibrational analyses of the compounds studied indicate an imperfect but fair correlation between the NO stretching frequency and NO(π*) occupancy, highlighting the usefulness of vibrational data as a preliminary indicator of the NO oxidation state.

## Abstract 2

Ab initio DMRG–CASSCF/CASPT2 calculations on iron porphyrin nitrosyl complexes have allowed clearer definition of the local oxidation state of the NO groups. The NO groups in both {FeNO}^6^ and {FeNO}^7^ porphyrins are found to be best described as NO^•^, while the {FeNO}^8^ anion [Fe(P)(NO)]^−^ (P = porphine anion) is best described as Fe(I)–NO^0^ ↔ Fe(II)–NO^−^. The calculations also provide valuable benchmarks for density functional theory vis-à-vis spin state energetics and spin densities.

## 1 Introduction

**Scheme 1:**
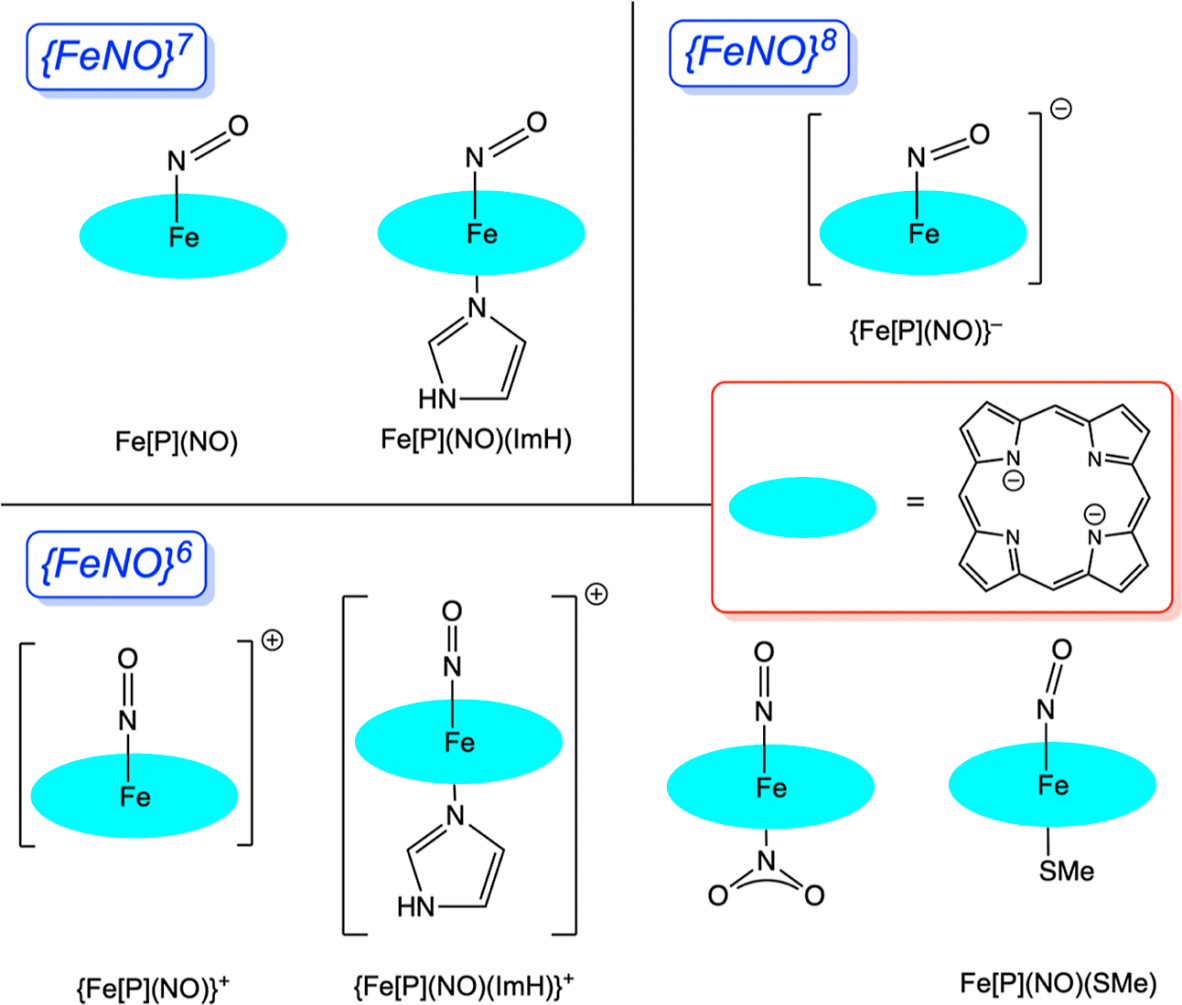
Molecules Studied in This Work

The electronic structures of transition metal nitrosyls have long been the subject of lively interest, debate, and controversy.^1−3^ The crux of the problem is that NO, as a paradigmatic noninnocent ligand, does not allow a simple determination of the oxidation state of a metal center it is attached to.^4,5^ According to current chemical nomenclature, oxidation states are defined in terms of the ionic approximation (IA), whereby the two electrons of a heteronuclear bond are both assigned to the more electronegative side.^6−8^ For NO complexes, the strongly covalent nature of metal(d)–NO(π*) interactions often interferes with the application of the IA. Fifty years ago, in a master stroke, Enemark and Feltham chose to sidestep the problem of local oxidation states by assigning an effective d electron count *n* to metal nitrosyls.^9^ Now known as the Enemark–Feltham electron count, *n* refers to the number of metal d electrons plus the number of NO π* electrons; thus, “Fe(II) + NO^•^” corresponds to *n* = 6 + 1 = 7 and is denoted as {FeNO}^7^. Despite the popularity of the notation, chemists have retained a strong interest in the oxidation state problem and have sought to assign oxidation states to both the metal and the NO fragments in nitrosyl complexes. Unfortunately, density functional theory, the major theoretical tool for such studies,^10−15^ suffers from several pitfalls. To start with, the DFT description generally does not correspond to a pure spin state but incorporates contamination from multiple states. In addition, different exchange–correlation functionals provide disturbingly divergent descriptions of metal–ligand covalence and of spin-state energetics.^16,17^ In the face of these challenges, chemists have increasingly resorted to a so-called spectroscopically calibrated approach, i.e., a combination of several spectroscopic methods and DFT calculations, to come up with local oxidation states in nitrosyl complexes.^18−23^ Modern multiconfigurational methods and orbital localization schemes provide an elegant alternative to these somewhat ad hoc approaches, as we illustrated recently in a study of transition metal corroles.^24^ Here we present a state-of-the-art DMRG/CASSCF–CASPT2 study of seven paradigmatic FeNO porphyrin derivatives spanning the {FeNO}^6–8^ electron counts (Scheme 1). Two {FeNO}^7^ systems were examined (see relevant experimental papers^25−31^): Fe[P](NO), i.e., a five-coordinate nitrosylheme, and its six-coordinate analogue Fe[P](NO)(ImH), where P is an unsubstituted porphyrin, and ImH is imidazole, a model for the amino acid histidine. Four oxidized {FeNO}^6^ systems, so-called met-heme nitrosyl derivatives, were examined (see relevant experimental papers^32−39^): (iii) {Fe[P](NO)}^+^, (iv) {Fe[P](NO)(ImH)}^+^, Fe[P](NO)(NO_2_), and Fe[P](NO)(SMe). Finally, one reduced {FeNO}^8^ system, a heme–nitroxide derivative, was examined (see relevant experimental papers^40−47^): {Fe[P](NO)}^−^. The present calculations provide a definitive resolution of several longstanding questions, including (i) the spin state energetics of the major classes of FeNO porphyrins, (ii) their spin density profiles (where applicable),^16,17^ and (iii) the local oxidation states of the Fe and the NO, as one transitions among Enemark–Feltham counts 6–8.

## 2 Methods

All structures, including excited states, were optimized with density functional theory employing the BP86 functional and def2-TZVP basis sets,^48−50^ with D3 dispersion corrections^51^ and Becke–Johnson damping.^52^ This method has been widely shown to yield realistic geometric structures for transition metal nitrosyls, such as in works by Conradie et al.^11^ and Monsch and Klüfers.^15^ Single-point calculations were carried out on these optimized geometries with a wide variety of exchange–correlation functionals (with different percentages of exact exchange shown in parentheses): PBE (0%), B97-D3 (0%), TPSS (0%), TPSSh (15%), B3LYP (20%), PBE0 (25%), BHLYP (25%), M06-L (0%), M06 (27%), and M06-2X (54%).

DMRG–CASSCF/CASPT2 calculations^53−61,81,96^ were performed with the OpenMolcas^62,63^ package interfaced with the CheMPS2 library.^64^ We used the aug-cc-pwCV5Z-DK basis set for Fe,^65^ cc-pVTZ-DK for H, and aug-cc-pVTZ-DK for the other ligand atoms,^66,67^ as we found that this combination gives the best agreement to the complete basis set limit due to error cancellations.^68^ Cholesky decomposition of the two-electron integrals with a threshold of 10^–6^ au was used.^69^ A second-order Douglas–Kroll–Hess (DKH) Hamiltonian^70−72^ was used to account for scalar relativistic effects. Similar to our previous works,^24,73^ the DMRG–CASSCF calculations made use of Fiedler orbital ordering,^74^ residual norm threshold of 10^–5^ for the Davidson algorithm, and perturbative noise with a prefactor of 0.05.^75^ We chose a value of 1000 for the number of renormalized states *m*, as it gave almost converged results in other studies on FeNO porphyrinoids.^76^ The ionization-potential/electron-affinity (IPEA) shift^77^ of 0.25 au and an imaginary shift^78^ of 0.1 au were used in the CASPT2 calculations. All core and semicore electrons of Fe (3s and 3p) were kept frozen in the CASPT2 treatment, as they make only a slight contribution to the CASPT2 relative energies in iron–nitrosyl complexes.^73^ Point group symmetry was employed, as appropriate.

The active spaces of the complexes are summarized in Table 1 and are similar to our previous work on nitrosyl complexes.^24^ The active spaces consist of all five Fe(3d) orbitals, all (possible) five Fe(4d) orbitals to account for the double-shell effect,^26^ all (possible) Fe-ligand σ orbitals, and a set of ten NO-based orbitals. The latter set includes two NO(π) and the correlating two NO(π*) orbitals; the NO(σ) orbital and the correlating NO(σ*) orbital, two NO(π′) orbitals to account for the radial correlation of the NO(π*) orbitals, one nitrogen 2s orbital, and the correlating orbital. The four Gouterman π orbitals (denoted Pπ) were also included, to allow for a noninnocent porphyrin in certain states (see Table 1). The natural active orbitals are shown in Figures S1–S4.

**Table 1:** Formal Electron Configuration of The Complexes Studied in This Work

electron configuration^a^	complex	active space^b^
[d_*xz*_ + π*(NO)]^2^ [d_*yz*_ + π*(NO)]^2^ (d_*xy*_)^↑^ (d_*x*^2^–*y*^2^_)^↑^ (d_*z*^2^_)^↑^	^4^{Fe[P](NO)}	19 in 23
	^4^{Fe[P](ImH)(NO)}	
[d_*xz*_ + π*(NO)]^2^ [d_*yz*_ + π*(NO)]^2^ (d_*xy*_)^2^ (d_*x*^2^–*y*^2^_)^0^ (d_*z*^2^_)^↑^	^2^{Fe[P](NO)}	19 in 22
	^2^{Fe[P](ImH)(NO)}	
[d_*xz*_ + π*(NO)]^2^ [d_*yz*_ + π*(NO)]^2^ (d_*xy*_)^2^ (d_*x*^2^–*y*^2^_)^0^ (d_*z*^2^_)^↑^ (Pπ)^↑^	^3^{Fe[P](NO)}^−^	22 in 23
[d_*xz*_ + π*(NO)]^2^ [d_*yz*_ + π*(NO)]^2^ (d_*xy*_)^2^ (d_*x^2^*–*y*^2^_)^0^ (d_*z*_^2^)^2^ (Pπ)^0^	^1^{Fe[P](NO)}^−^	22 in 23
[d_*xz*_ + π*(NO)]^2^ [d_*yz*_ + π*(NO)]^2^ (d_*xy*_)^↑^ (d_*x*^2^–*y*^2^_)^0^ (d_*z*^2^_)^↑^	^3^{Fe[P](NO)}^+^	18 in 22
	^3^{Fe[P] (NO)(ImH)}^+^	20 in 23
[d_*xz*_ + π*(NO)]^2^ [d_*yz*_ + π*(NO)]^2^ (d_*xy*_)^2^ (d_*x*^2^–*y*^2^_)^0^ (d_*z*^2^_)^0^	^1^{Fe[P](NO)}^+^	18 in 21
	^1^{Fe[P](NO)(ImH)}^+^	20 in 22
[d_*xz*_ + π*(NO)]^2^ [d_*yz*_ + π*(NO)]^2^ (d_*xy*_)^↑^ (d_*x*^2^–*y*^2^_)^0^ (d_*z*^2^_)^↑^	^3^{Fe[P](NO)(NO_2_)}	20 in 23
	^3^{Fe[P](NO)(SMe)}	
[d_*xz*_ + π*(NO)]^2^ [d_*yz*_ + π*(NO)]^2^ (d_*xy*_)^2^ (d_*x*^2^–*y*^2^_)^0^ (d_*z*^2^_)^0^	^1^{Fe[P](NO)(NO_2_)}	20 in 22
	^1^{Fe[P](NO)(SMe)}	

a Except for the case of linear FeNO, there is no clear distinction between d_*xz*_ and d_*z^2^*_ orbitals.

b We used the notation “*n*_e_ in *n*_a_” to denote an active space of *n*_e_ electrons in *n*_a_ active orbitals.

The DMRG–CASSCF wavefunctions were analyzed in terms of localized orbitals.^24,31,73,79^ All DMRG–CASSCF natural orbitals were first localized into ligand-based and Fe-based orbitals. We then used BLOCK2 to decompose the wave function into configuration state functions (CSFs).^80^ The CSFs were further classified into four resonance structures, Fe–NO^+^, Fe–NO^0^, Fe–NO^–^, and Fe–NO^2–^, allowing us to determine the oxidation state of Fe. We also examined the Mulliken spin populations calculated at the DMRG–CASSCF level of theory (see Supporting Information). As the DMRG–CASSCF interface in OpenMolcas lacks this functionality, the spin populations were calculated with the ORZ program package^81^ in combination with the def2-TZVP basis set.^50^ The formal electronic configurations of all complexes are shown in Table 1.

## 3 Results and Discussion

### 3.1 Spin State Energetics

Ever since density functional theory gained a widespread following among chemists, especially experimental chemists, the question of spin state energetics of transition metal complexes has been a vexing one.^82−87^ In early studies, we (as well as others) showed that classic pure functionals often exhibit an undue preference for lower-spin states, while hybrid functionals err in the opposite direction, favoring higher-spin states. In particular, we found the spin-crossover complex and nitrosylheme analogue Fe(salen)(NO)^16^ (salen = *N*,*N*′-bis(salicylidene)ethylenediamine; as well as other spin-crossover complexes^88−92^) to serve as a particularly useful test case for a functional’s performance vis-à-vis spin state energetics.

The CCSD(T) method has traditionally provided the gold standard for calculations of the spin state energetics of transition metal complexes. The DMRG–CASSCF/CASPT2 method employed here is slightly less accurate (with errors typically about 0.1–0.2 eV),^93−99^ but unlike CCSD(T) has the great advantage of applying to substantially multiconfigurational systems. For such systems, the DMRG–CASSCF/CASPT2 results can be calibrated by high-level multireference methods such as MR-ACPF and MR-ACQC.^100−104^ The latter methods are only applicable to small systems with only a few atoms, but these calculations afford reassuring calibration of CASPT2 energetics. Once again, the errors in the CASPT2 energetics are rarely worse than 0.1–0.2 eV. In the present study, we have tacitly assumed similar errors for adiabatic low-high spin-state gaps for a series of archetypal {FeNO}^6–8^ complexes. While worse than chemical accuracy, it is worth emphasizing that the scatter with different DFT functionals is about an order of magnitude higher. As of today, comparably accurate results are only available for Fe[P](NO)^31^ and Fe[C](NO),^24,73^ where P and C refer to unsubstituted porphine and corrole, respectively. Our main findings are as follows.

For the two {FeNO}^7^ complexes Fe[P](NO) and Fe[P](NO)(ImH), the DMRG–CASSCF/CASPT2 calculations predict a doublet ground state, as experimentally observed, and small doublet–quartet gaps (Δ*E*_DQ_ = *E*_quartet_ – *E*_doublet_) of 1–4 kcal/mol (Table 2). For comparison, common exchange–correlation functionals predict dramatic variations in Δ*E*_DQ_ values over a range spanning >40 kcal/mol. As expected, classic pure functionals greatly overstabilize the doublet state, whereas hybrid functionals with larger amounts of exact exchange incorrectly favor a quartet ground state by a wide margin. The popular hybrid functional B3LYP actually does rather well, yielding Δ*E*_DQ_ values in surprisingly good agreement with the DMRG–CASSCF/CASPT2 theory.

**Table 2:** Singlet–Triplet Gaps in {FeNO}^6^ and {FeNO}^8^ Porphyrins and the Doublet–Quartet Gaps in {FeNO}^7^ Porphyrins, Calculated with Various Functionals (Augmented with D3BJ Dispersion Corrections) and DMRG–CASPT2^a^

	{FeNO}^6^				{FeNO}^7^		{FeNO}^8^
	{Fe[P]NO}^+^	Fe[P](NO_2_)(NO)	{Fe[P](ImH)(NO)}^+^	Fe[P](SMe)NO	Fe[P]NO	Fe[P](ImH)(NO)	{Fe[P](NO)}^−^
BP86	12.7	20.8	22.2	15.4	18.4	18.4	6.4
PBE	13.0	21.0	23.3	15.7	17.9	19.7	6.3
B3LYP	8.1	9.2	20.2	3.5	0.1	3.8	3.8
TPSSh	10.6	13.7	_^b^	7.8	9.3	14.3	3.3
TPSS	12.9	18.9	23.8	13.4	18.7	21.4	5.7
BHLYP	7.7	–1.3	7.8	–8.9	–21.0	–18.8	3.3
PBE0	4.8	8.6	20.9	2.8	–5.5	–0.7	2.6
B97-D	9.8	19.1	15.6	14.1	5.8	3.5	6.6
M06	2.3	13.4	18.1	8.0	–12.3	–11.2	2.7
M06-L	8.5	17.2	21.5	12.9	–1.3	3.3	4.1
M06-2X	–2.8	–0.9	5.6	–7.6	–24.4	–25.0	3.7
DMRG–CASPT2	20.9	33.6	30.4	31.2	1.0	3.6	20.7

a All values are in kcal/mol.

b Calculation did not converge to the correct state.

Somewhat to our surprise, DMRG–CASSCF/CASPT2 calculations predict surprisingly large singlet–triplet gaps of >30 kcal/mol for the three {FeNO}^6^ complexes {Fe[P](NO)(ImH)}^+^, Fe[P](NO)(NO_2_) and Fe[P](NO)(SMe). This gap also appears to be relatively independent of the axial ligand. The latter observation is surprising in that the axial thiolate and nitrite ligands are both readily oxidized as independent species and, naively speaking, a low-energy, antiferromagnetically coupled {FeNO}^7^–L^•^ ligand radical state might have been expected (as was indeed speculated by Walker^34^), in stark contrast to the DMRG–CASSCF/CASPT2 results. For these complexes, most of the exchange–correlation functionals perform qualitatively well, correctly indicating singlet ground states but generally underestimating the singlet–triplet gap (Δ*E*_ST_ = *E*_triplet_ – *E*_singlet_). Once again, the functionals with the highest proportions of exact exchange fail to identify the correct ground state, i.e., incorrectly predict a triplet ground state.

For the {FeNO}^8^ complex {Fe[Por](NO)}^−^, DMRG–CASSCF/CASPT2 calculations predict an unambiguous singlet ground state and a high singlet–triplet gap of >20 kcal/mol, qualitatively mirroring the scenario obtained for the {FeNO}^6^ complexes. For {Fe[Por](NO)}^−^, however, all exchange–correlation functionals correctly predict a singlet ground state, but with much smaller Δ*E*_ST_’s relative to the DMRG–CASSCF/CASPT2 theory.

### 3.2 Spin Density Profiles

**Figure 1:**
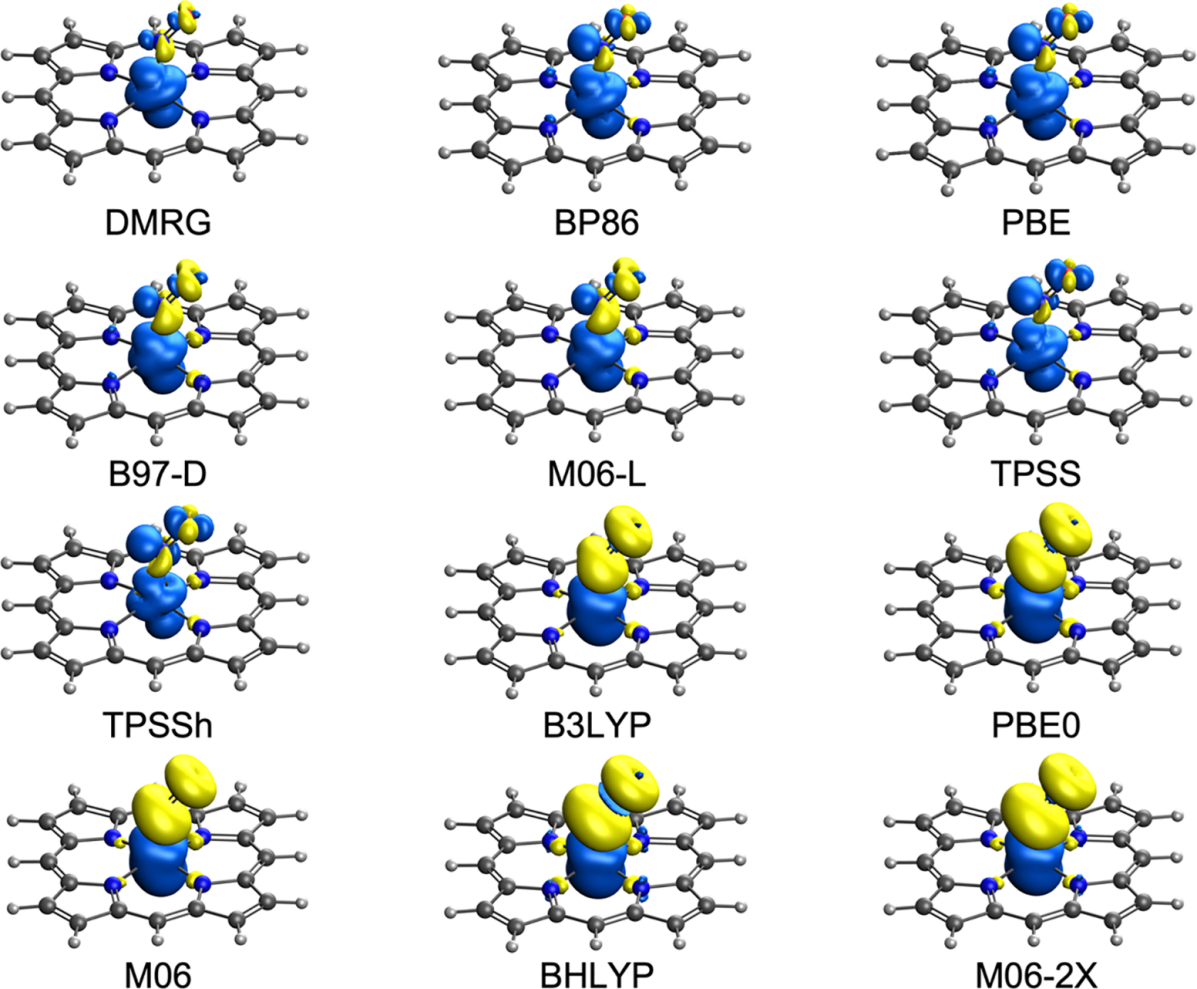
DMRG–CASSCF and DFT spin density plots of ^2^Fe[P](NO), with majority and minority spin densities colored blue and yellow, respectively.

**Figure 2:**
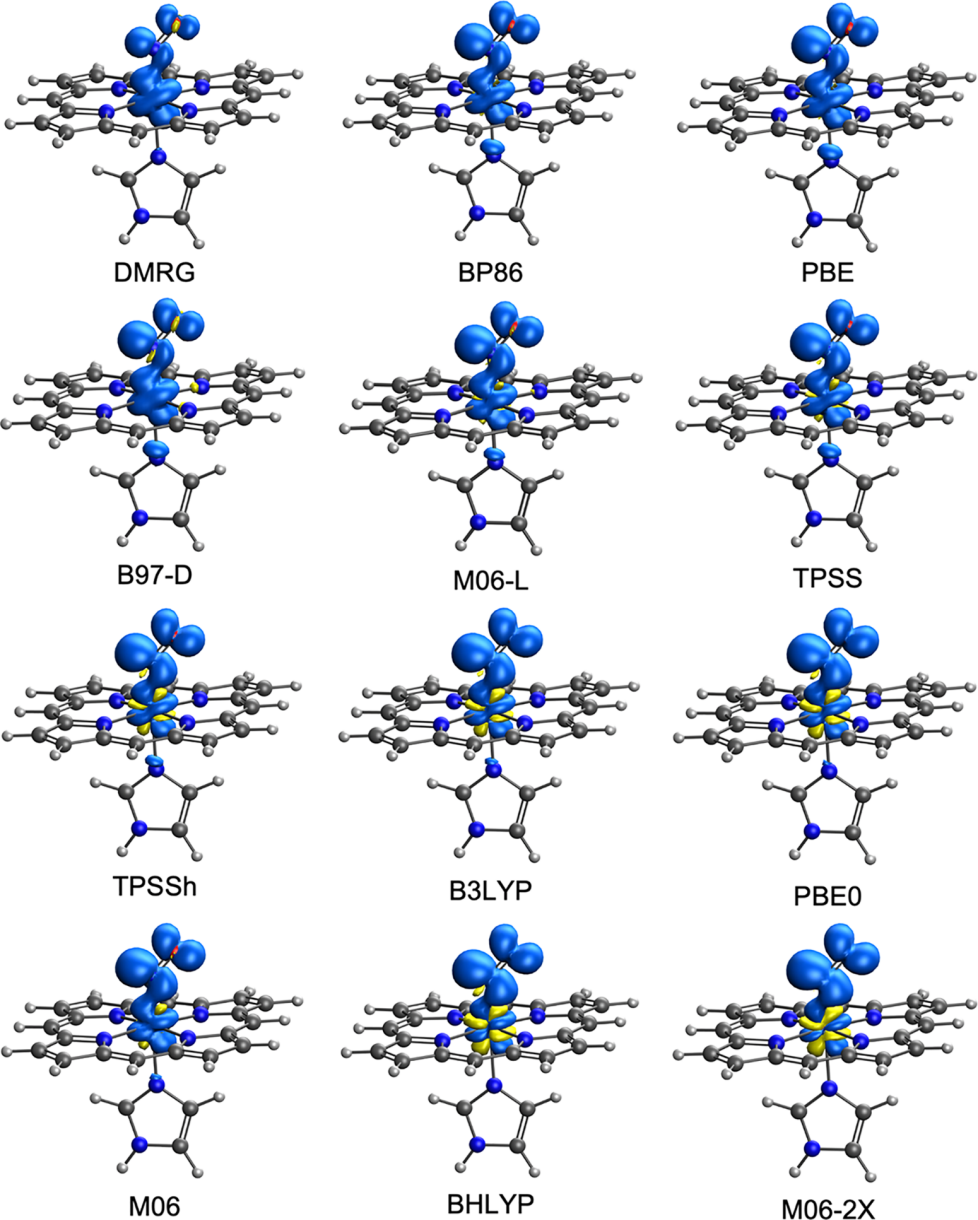
DMRG–CASSCF and DFT spin density plots of ^2^Fe[P](NO)(ImH), with majority and minority spin densities colored blue and yellow, respectively.

DMRG–CASSCF calculations predict that nearly the entire spin density in Fe[Por](NO) is localized on the Fe with only a trace on the NO. In Fe[Por](NO)(ImH), the Fe carries about four-fifths of the spin density, with most of the remaining fifth on the NO, reflecting the effect of the antibonding Fe(d_*z*^2^_)–ImH antibonding interaction. As shown in Figures 1 and 2, pure functionals largely capture the essence of the DMRG spin density profile, whereas hybrid functionals lead to much greater spatial separation of the majority and minority (alternatively, up and down) spin densities. For the singlet {FeNO}^6^ and {FeNO}^8^ species, DMRG–CASSCF calculations “by definition” indicate zero spin density at every point, in contrast to DFT, which results in various degrees of spin symmetry-breaking, from negligible for classic pure functionals to pronounced for hybrid functionals. The fact that the large Fe spin density in the {FeNO}^7^ state is neutralized in the {FeNO}^6^ and {FeNO}^8^ states may be naively regarded as indicative of essentially metal-centered oxidation and reduction, respectively. It is worth recalling that early UV–vis spectroelectrochemical studies of simple {FeNO}^7^ porphyrins by Kadish and co-workers also reached similar conclusions, i.e., FeNO-centered redox processes.^40^ Below we shall see that an analysis of the DMRG wave function adds considerable detail to these qualitative arguments.

### 3.3 DMRG–CASSCF Resonance Structures and Implications for Oxidation States

**Figure 3:**
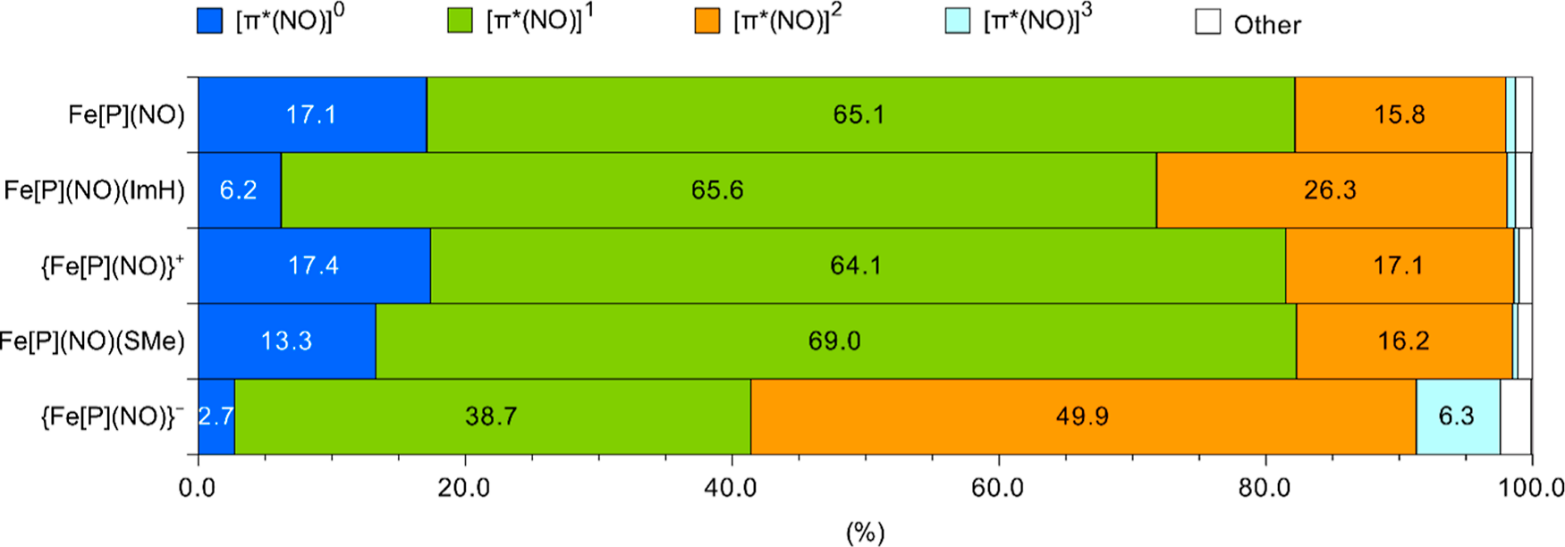
Weights (in percentage) of dominant configurations based on (NO–π*)^*n*^ (*n* = 0, 1, 2, 3) in DMRG–CASSCF wave functions, indicated in different colors. The localization procedure fails to localize the NO–π orbitals and σ orbital between Fe and the axial ligand in {Fe[P](NO)(ImH)}^+^ and Fe[P](NO)(NO_2_).

As explained above in Methods Section, we decomposed the DMRG–CASSCF wave function into “resonance forms” in which the total NO π*-occupancy varies from 0 to 4; the results are shown in Figure 3. Note that this analysis does not directly yield an oxidation state for the Fe or NO, but identifies resonance forms in order of importance. It is the latter that provides the basis for a discussion of oxidation states. One drawback of this approach is that the localization procedure may fail for certain species, as it did for the {FeNO}^6^ complexes {Fe[P](NO)(ImH)}^+^ and Fe[P](NO)(NO_2_). Fortunately, the method worked satisfactorily for the other two {FeNO}^6^ complexes studied, allowing for a comparative discussion of all three Enemark–Feltham electron counts of interest in this study.

For both of the {FeNO}^7^ complexes examined, Fe[P](NO) and Fe[P](NO)(ImH), approximately two-thirds of the wave function is made up of [π*(NO)]^1^ configurations, with the remaining third made up of a mix of [π*(NO)]^0^ and [π*(NO)]^2^ configurations. The axial imidazole ligand decreases the proportion of [π*(NO)]^0^ configurations and increases that of [π*(NO)]^2^ configurations, while leaving the proportions of [π*(NO)}^1^ configurations relatively unaffected. This finding mirrors the impact of the imidazole ligand on the spin density profile of Fe[P](NO). Thus, in spite of the minor difference, both complexes can, to a first approximation, be described as Fe(II)–NO^0^. It is worth stressing that this analysis does not imply that the NO ligand in these two complexes carries a large or even significant amount of electronic spin density.

For the two {FeNO}^6^ complexes analyzed, [π*(NO)]^1^ configurations also account for approximately two-thirds of the wave function, with the remaining third made up of a roughly even mix of [π*(NO)]^0^ and [π*(NO)]^2^ configurations. Given that the porphyrin is thought to be innocent with a formal charge of −2 in all the complexes, we may, accordingly, at least to a first approximation, describe the two complexes as Fe(III)–NO^0^. Such a description is at variance with from the popular view of low-spin, square-pyramidal or octahedral {FeNO}^6^ complexes as Fe(II)–NO^+^,^2,19^ but is consonant with Solomon^21^ and co-workers’ L-edge X-ray absorption study of an octahedral nonheme {FeNO}^6^ complex with “heme-like” coordination.^105^ Another study by DeBeer, Meyer, and co-workers^20^ has also reached a similar conclusion.

In {Fe[P](NO)}^−^, the contribution of [π*(NO)}^1^ configurations is dramatically lower, with that of the [π*(NO)}^2^ configurations correspondingly higher. Accordingly, to a first approximation, {Fe[P](NO)}^−^ appears best described as a resonance hybrid: Fe(I)–NO^0^ ↔ Fe(II)–NO^–^. Going from {FeNO}^7^ to {FeNO}^8^, the reduction thus is not entirely metal-centered, as speculated above, but also significantly on the NO. Such a description is largely in accord with earlier theoretical studies on low-spin {FeNO}^8^ species,^42,45^ including one by one of us.^43^

### 3.4 Insights from NO Bond Distances and Vibrational Frequencies

Given that the NO bond distance and vibrational frequency are known to vary as a function of the NO π* occupancy, we looked into the possibility of a semiquantitative correlation. Toward that end, we optimized and determined the vibrational frequency of NO as an isolated diatomic, with the π* occupation varying from 0 to 2 (i.e., from NO^+^ to NO^–^). Fractional orbital occupations were also employed in this exercise. An essentially linear relationship was found to exist among the N–O distance, vibrational frequency, and π* occupancy. As hoped for, the N–O distances and vibrational frequencies of the FeNO porphyrins studied also appeared to follow the same relationship, allowing an empirical readout of NO π* occupancies in the different molecules (Figure 3). Note that the couplings between the NO vibration and other vibrational modes are small. In all complexes, the NO bond distance ranges from 1.153 to 1.203 Å, but never exceeds the value of 1.213 Å corresponding to NO^–0.5^ [or the occupancy of 1.5 of the NO(π*) orbitals]. Similarly, the NO vibrational frequency ranges from 1529 to 1945 cm^–1^, corresponding to somewhat under NO^–0.5^ (1635 cm^–1^) to somewhat over NO^0^ (1889 cm^–1^). Overall, the results indicate that the vast majority of the complexes, regardless of their spin state, are best described as metal–NO^0^ as opposed to metal–NO^–^ or metal–NO^+^.

**Figure 4:**
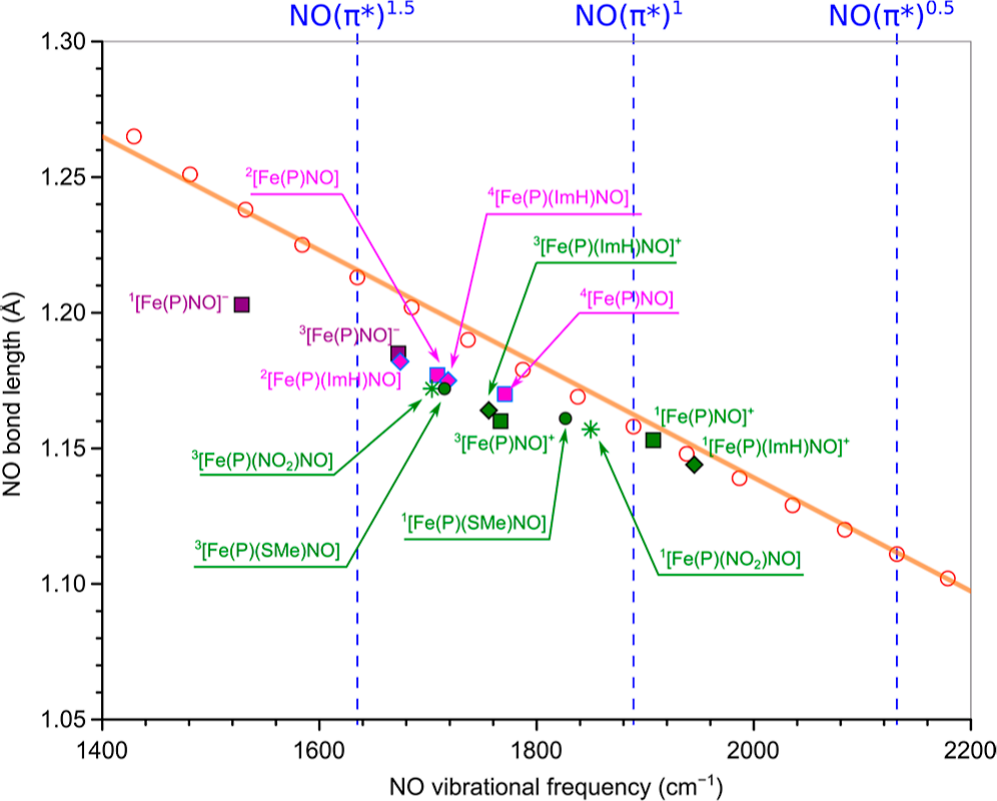
Correlation between the NO bond distance and vibrational frequency, obtained with the BP86-D3(BJ)/def2-TZVP method. The open-circles correspond to the results of isolated NO with fractional orbital occupations.

Using the calibration curve, one can also estimate the π* occupancies of the complexes, although the results should be viewed qualitatively, as we found a significant downshift of the data points from the calibration curve. This behavior is also found in other nonheme complexes but to a smaller extent (unpublished results). Based on the NO vibrational frequency, the occupancies should be 0.95, 1.35, and 1.7 for {Fe[P](NO)}^+^, Fe[P](NO), and {Fe[P](NO)}^−^, respectively. However, based on the NO bond distance, the occupancies are 0.90, 1.15, and 1.4, respectively. These results are in moderate agreement with those obtained via the DMRG–CASSCF-based resonance form analysis outlined above. The analysis suggests that the NOs in both {Fe[P](NO)}^+^ and Fe[P](NO) are best approximated as NO^0^, while the one in {Fe[P](NO)}^−^ is around NO^–0.5^. On the other hand, this analysis is inconsistent with the result that both {Fe[P](NO)}^+^ and Fe[P](NO) exhibit a nearly identical NO resonance form composition, as shown in Figure 3. From the point of view of oxidation state assignment, we view resonance form analysis as the clearly superior method. The diatomic model that forms the basis of Figure 4 is clearly a gross oversimplification of the dynamics of the FeNO group.

## 4 Conclusions

High-level ab initio DMRG–CASSCF/CASPT2 calculations on archetypal {FeNO}^6^, {FeNO}^7^, and {FeNO}^8^ heme–nitrosyl complexes have yielded a number of new insights as well as underscored significant deficiencies of DFT methods. The key results are enumerated as follows.

(a) As a result of the balanced treatment of static and dynamic correlation, DMRG–CASSCF/CASPT2 calculations have provided some of the most authoritative results available to date on the spin state energetics of heme–nitrosyl complexes. DFT calculations, in contrast, yield widely divergent results on spin state energetics as a function of the exchange–correlation functional, even though the various functionals correctly identify the ground states of transition metal complexes for the great majority of transition metal complexes. As far as spin state energetics is concerned, DMRG–CASSCF/CASPT2 calculations indicate that (a) {FeNO}^7^ complexes, represented by Fe[P](NO) and Fe[P](ImH)(NO), exhibit small doublet–quartet gaps, typically ≲4 kcal/mol, and (b) both {FeNO}^6^ and {FeNO}^8^ complexes exhibit large singlet–triplet gaps of ≳20 kcal/mol. In other words, the Fe–NO bonding in the latter two classes of complexes is strongly covalent and should not be described as antiferromagnetic coupling.
(b) DMRG–CASSCF spin densities have provided valuable benchmarks for those obtained with DFT. Thus, DMRG–CASSCF calculations predict nearly the entire spin density of Fe[P](NO) localized on the iron, whereas, in the case of Fe[P](NO)(ImH), the sixth ligand pushes approximately a fifth of that spin density out on to the NO. These spin density patterns are similar to those obtained with pure functionals, but quite different from those obtained with hybrid functionals. The latter exhibit with much greater separation of majority and minority spin densities, reflecting contamination from the *S* = 3/2 state.
(c) An analysis of the DMRG–CASSCF wave function in terms of localized orbitals has permitted a quantitative assessment of the contributions of resonance forms with different NO(π*) occupancies, i.e., especially the metal–NO^+^, metal–NO^0^, metal–NO^–^, and metal–NO^2–^ resonance forms. For the {FeNO}^7^ and {FeNO}^6^ complexes studied, the wave function in each case indicated a dominant NO^0^ resonance form. For the {FeNO}^8^ complex {Fe[P](NO)}^−^, a similar exercise indicated a resonance hybrid, Fe(I)–NO^0^ ↔ Fe(II)–NO^–^, with both resonance forms making comparable contributions of 44 ± 6%. These findings contradict a number of common formulations for nitrosyl complexes, most notably Fe(II)–NO^+^ for {FeNO}^6^ heme–nitrosyl systems, but are consonant with an L-edge XAS study of an octahedral low-spin nonheme {FeNO}^6^ complex, which the authors formulated as Fe(III)–NO^0^. To what extent the present conclusions are transferable to high-spin nonheme iron nitrosyls remains a fascinating question at this point.

We wish to conclude by reaffirming our continued support and admiration for the 50-year-old Enemark–Feltham formalism. Far from being a “cop-out” in terms of ducking the question of local oxidation states, it is a much-needed reminder of the complex multiconfigurational character of transition metal nitrosyls.

## Data Availability

All data generated or analyzed in this study are included in this published article and its Supporting Information.

## Supporting Information Available

Optimized Cartesian coordinates of the complexes; Mulliken spin population values calculated with different exchange–correlation functionals and DMRG–CASSCF theory; and active orbitals in DMRG–CASSCF calculations (PDF)
