# Supplementary material for: Local Oxidation States in {FeNO}6–8 Porphyrins: Insights from DMRG/CASSCF–CASPT2 Calculations
Source: Inorg Chem. 2023 Nov 27;62(49):20496–505. doi: 10.1021/acs.inorgchem.3c03689 (PMC10716898; doi:10.1021/acs.inorgchem.3c03689)
Supplement: Supplementary file 1 — ic3c03689_si_001.pdf [file ic3c03689_si_001.pdf]

Supporting Information

Local Oxidation States in {FeNO}$^{6–8}$ Porphyrins: Insights from DMRG/CASSCF-CASPT2 Calculations

Quan Manh Phung*[,a,b], Ho Ngoc Nam[c,d], and Abhik Ghosh*[,e]

[a] Department of Chemistry, Graduate School of Science, Nagoya University, Furo-cho, Chikusa-ku, Nagoya, Aichi 464-8602, Japan

[b] Institute of Transformative Bio-Molecules (WPI-ITbM), Nagoya University, Furo-cho, Chikusa-ku, Nagoya, Aichi, 464-8601, Japan

[c] Institute of Materials Innovation, Institutes of Innovation for Future Society, Nagoya University, Furo-cho, Chikusa-ku, Nagoya, Aichi 464-8601, Japan

[d] Department of Chemical Systems Engineering, Graduate School of Engineering, Nagoya University, Furo-cho, Chikusa-ku, Nagoya, Aichi 464-8603, Japan

[e] Department of Chemistry, UiT The Arctic University of Norway, N-9037 Tromsø, Norway

Email: quan.phung@chem.nagoya-u.ac.jp (QMP); abhik.ghosh@uit.no (AG)

We have used the $^{2S+1}$\{complex\}^{c} notation throughout the study, where $2S+1$ is the spin multiplicity and c is the molecular charge.

List of Figures

Figure S1. Active orbitals in Fe[P](NO), {Fe[P](NO)}$^{-}$, and {Fe[P](NO)}$^{+}$ complexes. 3
Figure S2. Active orbitals in Fe[P](ImH)(NO) and {Fe[P](ImH)(NO)}$^{+}$ complexes. 4
Figure S3. Active orbitals in Fe[P](NO_2)(NO). 5
Figure S4. Active orbitals in Fe[P](SMe)(NO)...6

List of Tables

Table S1. Spin population values in ^{4}{Fe[P](NO)}, calculated with different functionals and DMRG 7
Table S2. Spin population values in ^{2}{Fe[P](NO)}, calculated with different functionals and DMRG 7
Table S3. Spin population values in ^{3}{Fe[P](NO)}$^{+}$, calculated with different functionals and DMRG....8
Table S4. Spin population values in ^{1}{Fe[P](NO)}$^{+}$, calculated with different functionals and DMRG....8
Table S5. Spin population values in ^{3}{Fe[P](NO)}$^{-}$, calculated with different functionals and DMRG....9
Table S6. Spin population values in ^{1}{Fe[P](NO)}$^{-}$, calculated with different functionals and DMRG....9
Table S7. Spin population values in ^{4}{Fe[P](ImH)(NO)}, calculated with different functionals and DMRG...10
Table S8. Spin population values in ^{2}{Fe[P](ImH)(NO)}, calculated with different functionals and DMRG...10
Table S9. Spin population values in ^{3}{Fe[P](ImH)(NO)}$^{+}$, calculated with different functionals and DMRG...11
Table S10. Spin population values in ^{1}{Fe[P](ImH)(NO)}$^{+}$, calculated with different functionals and DMRG...11
Table S11. Spin population values in ^{3}{Fe[P](NO_2)(NO)}, calculated with different functionals and DMRG...12
Table S12. Spin population values in ^{1}{Fe[P](NO_2)(NO)}, calculated with different functionals and DMRG...12
Table S13. Spin population values in ^{3}{Fe[P](SMe)(NO)}, calculated with different functionals and DMRG...13
Table S14. Spin population values in ^{1}{Fe[P](SMe)(NO)}, calculated with different functionals and DMRG...13
Table S15. Selected bond distances^{c} (in Å), Fe−NO bond angle (in degree), and NO vibrational wavenumber (in cm^{-1})...14
Table S16. Corelation between charge, vibrational frequency, and bond length of NO.......................15

Active orbitals

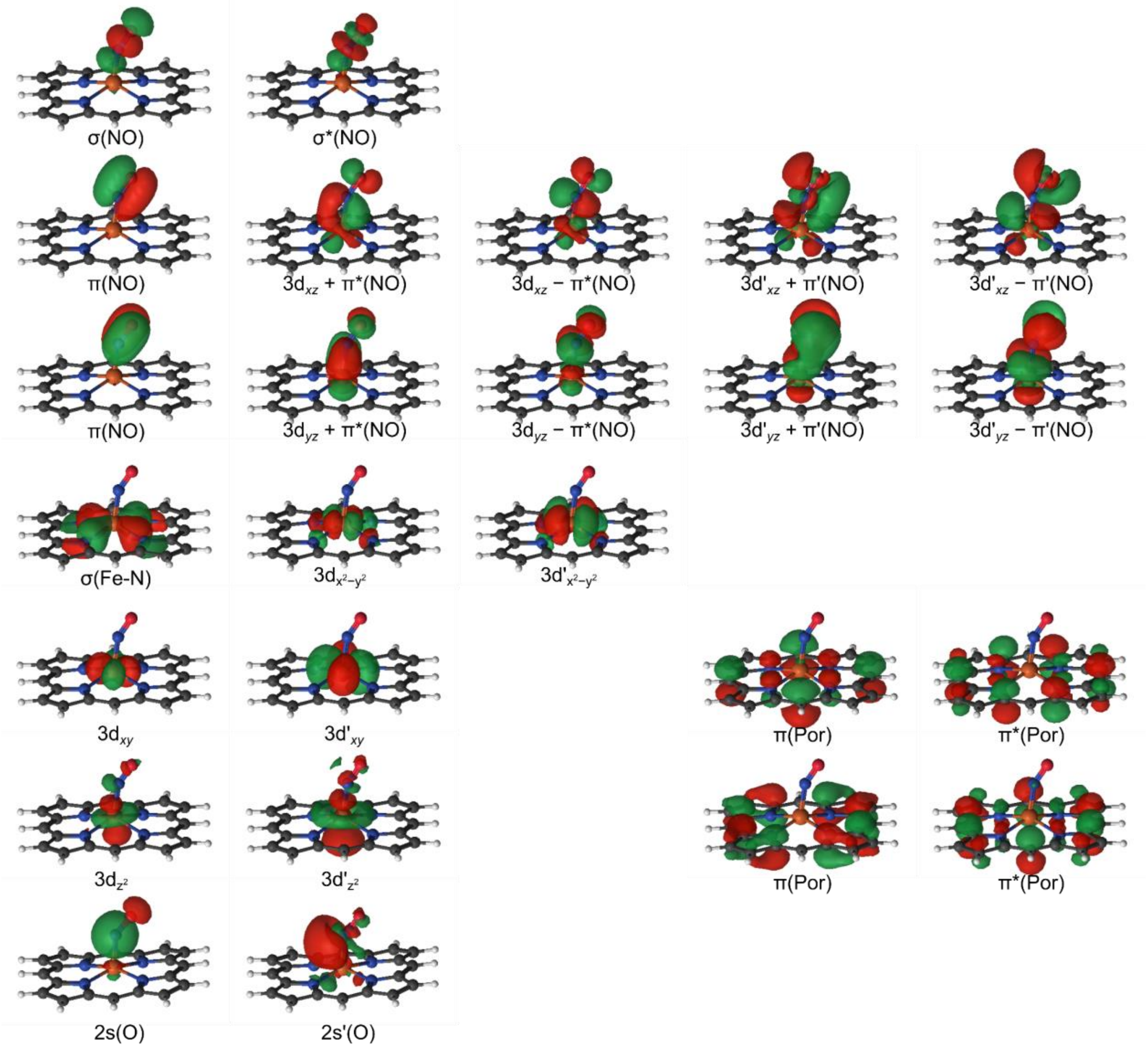

Figure S1. Active orbitals in Fe[P](NO), {Fe[P](NO)}$^{-}$, and {Fe[P](NO)}$^{+}$ complexes. In ^{4}{Fe[P](NO)}, 2s(O) and 2s'(O) are neglected. In ^{2}{Fe[P](NO)} and ^{3}{Fe[P](NO)}$^{+}$, 2s(O), 2s'(O), $3d'_{x2-y2}$ are neglected. In ^{3}{Fe[P](NO)}$^{-}$ and ^{1}{Fe[P](NO)}$^{-}$, $3d'_{x2-y2}$, and $3d'_{z2}$ are neglected. In ^{1}{Fe[P](NO)}$^{+}$, 2s(O), 2s'(O), $3d'_{x2-y2}$, and $3d'_{z2}$ are neglected.

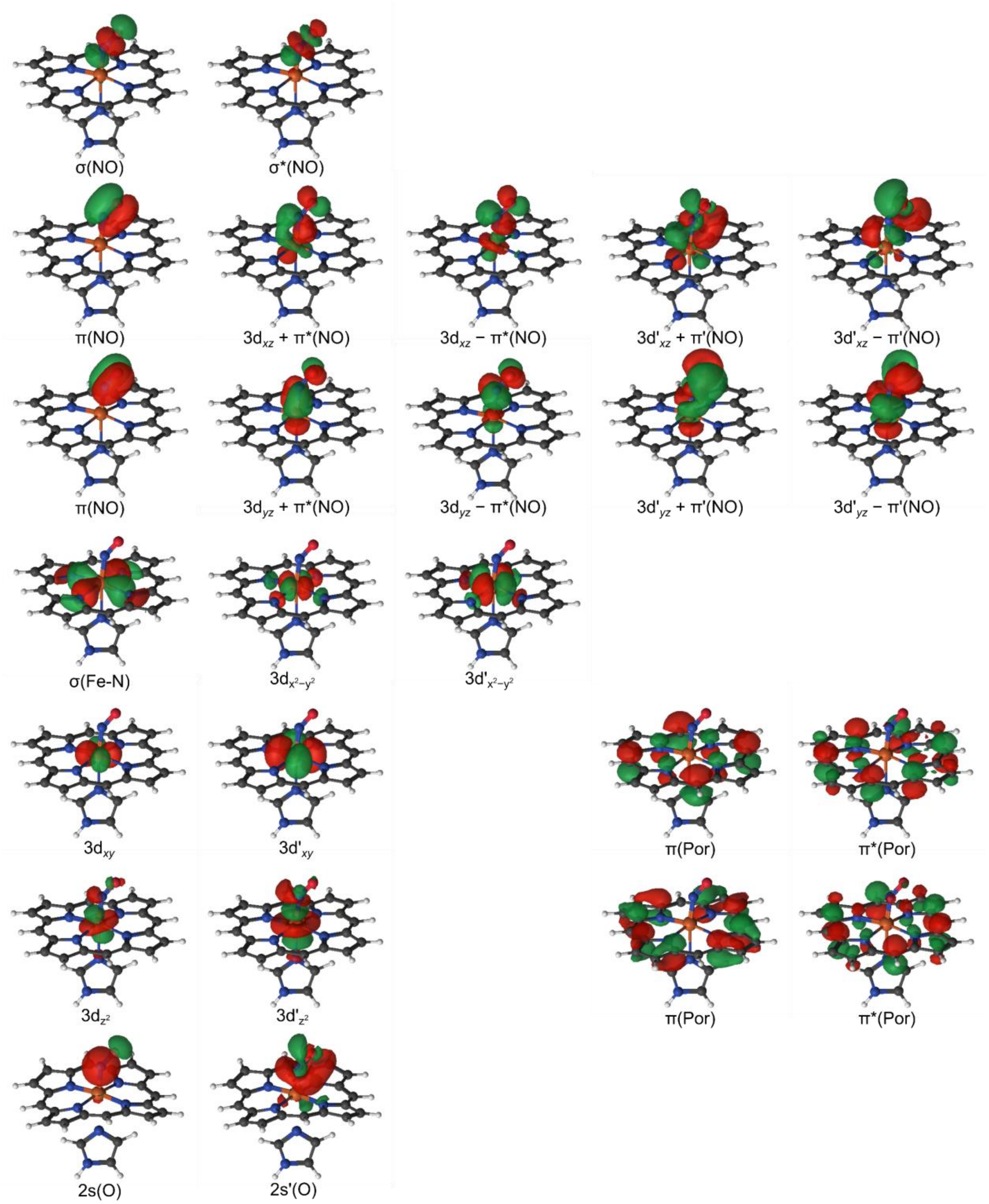

Figure S2. Active orbitals in Fe[P](ImH)(NO) and $\{Fe[P](ImH)(NO)\}^+$ complexes. In $^4\{Fe[P](ImH)(NO)\}$, 2s(O) and 2s'(O) are neglected. In $^2\{Fe[P](ImH)(NO)\}$, 2s(O), 2s'(O), 3d'$_{x2-y2}$ are neglected. In $^3\{Fe[P](ImH)(NO)\}^+$, 3d'$_{x2-y2}$, and 3d'$_{z2}$ are neglected. In $^1\{Fe[P](ImH)(NO)\}^+$, 2s'(O), 3d'$_{x2-y2}$, and 3d'$_{z2}$ are neglected.

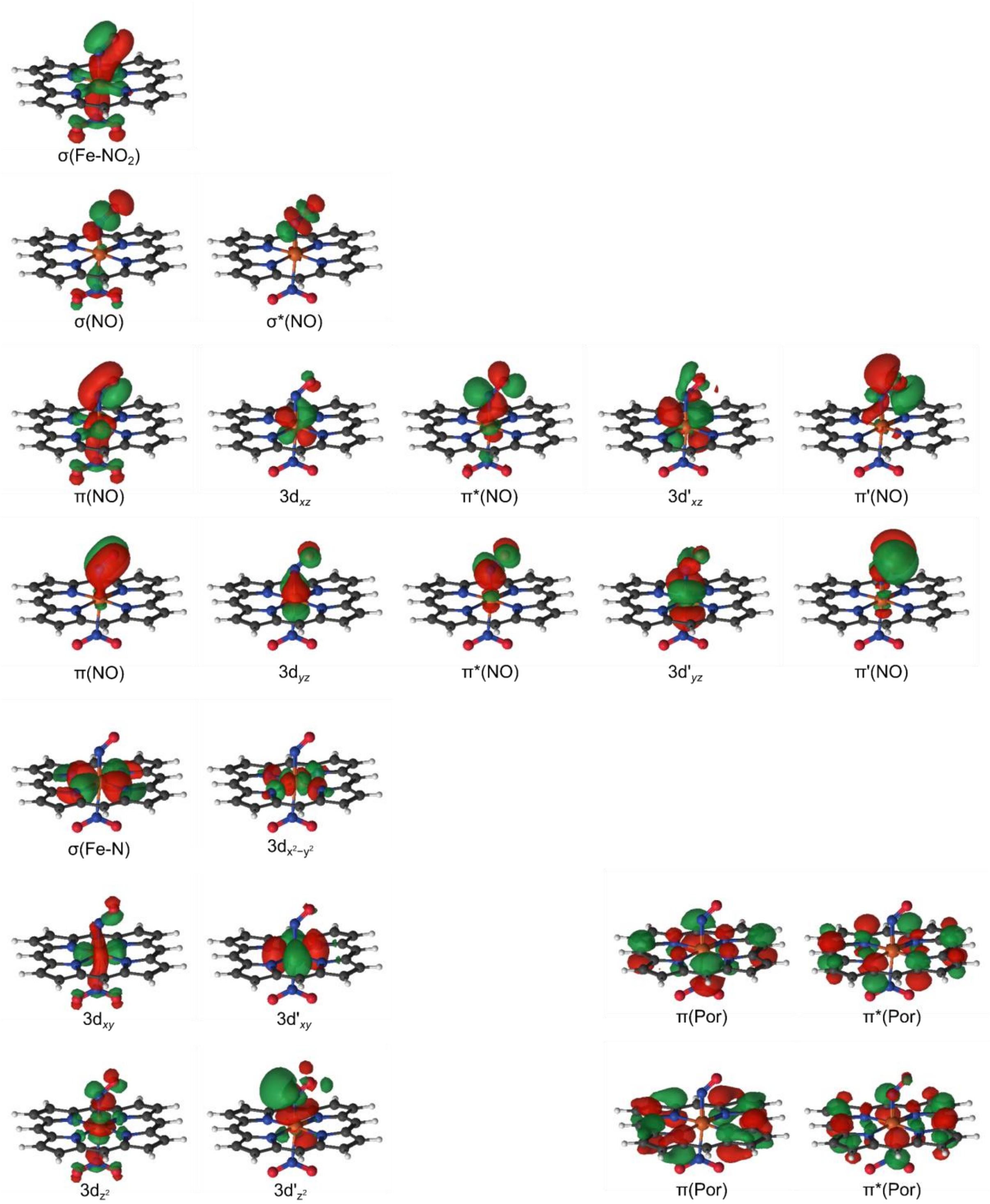

Figure S3. Active orbitals in Fe[P](NO_2)(NO). In ^{1}{Fe(P)(ImH)(NO)}, 3d'$_{z2}$ is neglected.

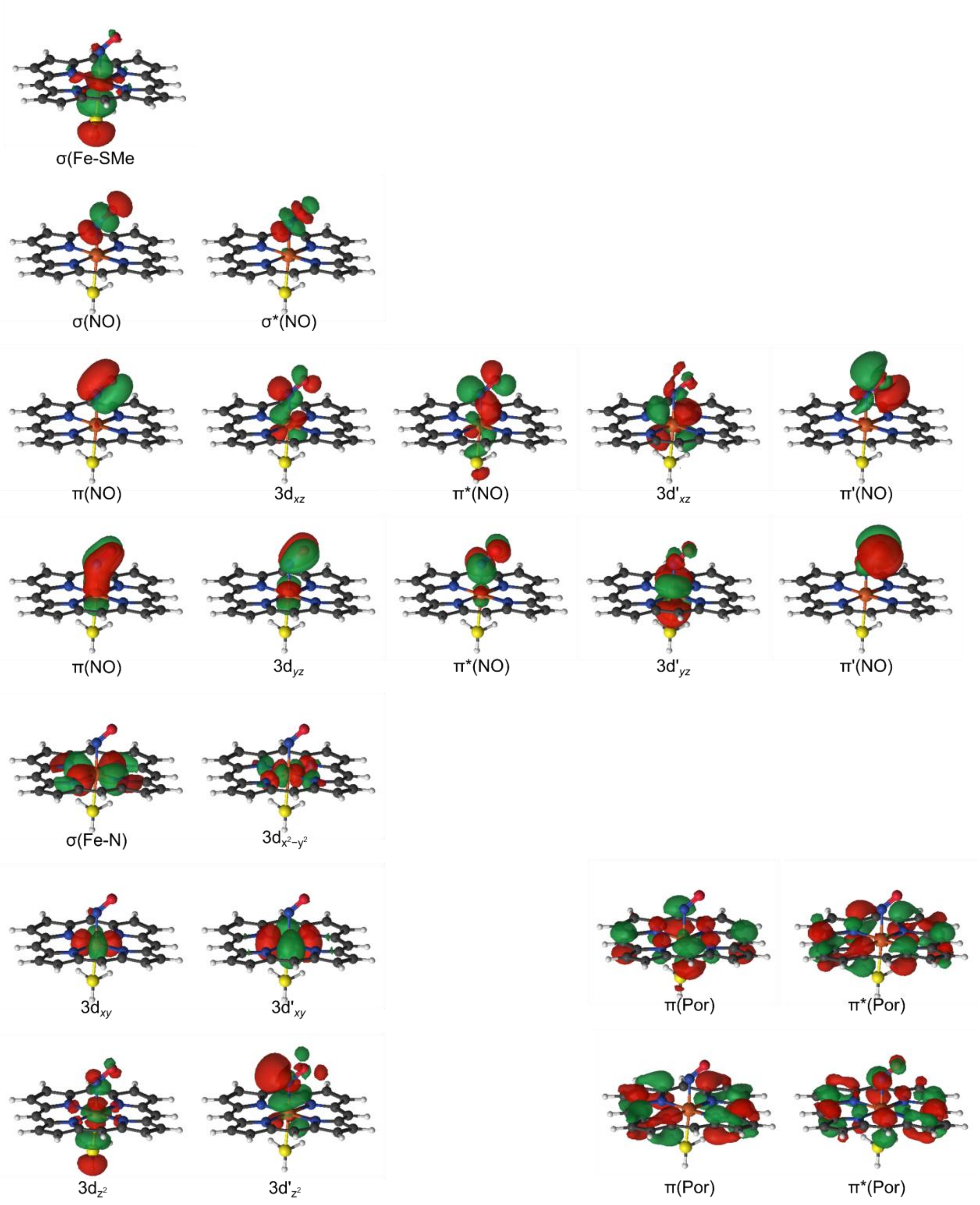

Figure S4. Active orbitals in Fe[P](SMe)(NO)]. In ^{1}{Fe[P](SMe)(NO)}, $3d'_{z2}$ is neglected.

Mulliken Spin Population Analysis

Table S1. Spin population values in ^{4}{Fe[P](NO)}, calculated with different functionals and DMRG

Complex	Fe	N	O	NO	The rest
BP86	3.30	−0.34	−0.30	−0.64	0.34
PBE	3.28	−0.33	−0.30	−0.63	0.35
B3LYP	3.75	−0.56	−0.52	−1.09	0.34
TPSSh	3.65	−0.51	−0.45	−0.95	0.30
TPSS	3.36	−0.37	−0.32	−0.69	0.33
BHLYP	4.11	−0.72	−0.69	−1.41	0.30
PBE0	3.89	−0.61	−0.57	−1.17	0.29
B97−D	3.44	−0.43	−0.38	−0.81	0.37
M06	3.81	−0.58	−0.54	−1.12	0.31
M06−L	3.67	−0.51	−0.47	−0.98	0.30
M06−2X	4.01	−0.64	−0.69	−1.33	0.33
DMRG	3.38	−0.30	−0.23	−0.53	0.15

Table S2. Spin population values in ^{2}{Fe[P](NO)}, calculated with different functionals and DMRG

Complex	Fe	N	O	NO	The rest
BP86	0.94	0.07	0.00	0.07	−0.01
PBE	0.94	0.07	0.00	0.07	−0.01
B3LYP	1.60	−0.27	−0.29	−0.55	−0.05
TPSSh	0.95	0.08	0.00	0.08	−0.03
TPSS	0.90	0.10	0.02	0.12	−0.02
BHLYP	2.38	−0.68	−0.64	−1.32	−0.06
PBE0	1.98	−0.45	−0.44	−0.89	−0.09
B97−D	1.11	−0.02	−0.07	−0.09	−0.02
M06	1.86	−0.40	−0.40	−0.80	−0.06
M06−L	1.18	−0.04	−0.08	−0.12	−0.06
M06−2X	2.24	−0.58	−0.65	−1.23	−0.01
DMRG	1.03	−0.02	−0.02	−0.04	0.01

Table S3. Spin population values in $^{3}\{Fe[P](NO)\}^{+}$, calculated with different functionals and DMRG

Complex	Fe	N	O	NO	The rest
BP86	1.68	−0.02	−0.06	−0.08	0.40
PBE	1.68	−0.01	−0.06	−0.07	0.39
B3LYP	2.48	−0.21	−0.27	−0.48	0.01
TPSSh	1.75	−0.04	−0.08	−0.11	0.37
TPSS	1.60	0.00	−0.03	−0.03	0.43
BHLYP	3.06	−0.45	−0.36	−0.80	−0.26
PBE0	2.83	−0.31	−0.38	−0.69	−0.14
B97−D	1.96	−0.14	−0.16	−0.29	0.34
M06	2.85	−0.34	−0.40	−0.75	−0.10
M06−L	2.29	−0.21	−0.24	−0.45	0.16
M06−2X	3.00	−0.39	−0.42	−0.81	−0.19
DMRG	2.15	−0.08	−0.07	−0.15	0.00

Table S4. Spin population values in $^{1}\{Fe[P](NO)\}^{+}$, calculated with different functionals and DMRG

Complex	Fe	N	O	NO	The rest
BP86	0.04	0.00	0.00	0.00	−0.04
PBE	0.03	0.00	0.00	0.00	−0.03
B3LYP	1.06	−0.27	−0.29	−0.56	−0.50
TPSSh	0.39	−0.08	−0.08	−0.16	−0.22
TPSS	0.06	0.00	0.00	0.00	−0.06
BHLYP	2.19	−0.56	−0.63	−1.19	−1.01
PBE0	1.38	−0.35	−0.38	−0.73	−0.65
B97−D	0.07	−0.01	−0.01	−0.03	−0.04
M06	1.22	−0.29	−0.32	−0.61	−0.61
M06−L	0.34	−0.05	−0.06	−0.11	−0.22
M06−2X	2.05	−0.48	−0.64	−1.12	−0.93
DMRG	0.00	0.00	0.00	0.00	0.00

Table S5. Spin population values in $^{3}\{Fe[P](NO)\}^{-}$, calculated with different functionals and DMRG

Complex	Fe	N	O	NO	The rest
BP86	0.95	0.06	0.00	0.06	0.99
PBE	1.75	−0.36	−0.34	−0.70	0.95
B3LYP	0.99	0.06	0.00	0.06	0.95
TPSSh	0.90	0.10	0.02	0.12	0.99
TPSS	2.42	−0.74	−0.63	−1.37	0.94
BHLYP	2.04	−0.50	−0.46	−0.96	0.91
PBE0	1.12	−0.03	−0.07	−0.11	0.98
B97−D	1.89	−0.43	−0.40	−0.83	0.94
M06	1.23	−0.07	−0.10	−0.17	0.94
M06−L	2.29	−0.64	−0.65	−1.29	1.00
M06−2X	0.92	0.07	0.01	0.08	1.00
DMRG	0.95	0.06	0.00	0.06	0.99

Table S6. Spin population values in $^{1}\{Fe[P](NO)\}^{-}$, calculated with different functionals and DMRG

Complex	Fe	N	O	NO	The rest
BP86	0.21	−0.03	−0.02	−0.05	−0.16
PBE	0.21	−0.03	−0.02	−0.05	−0.16
B3LYP	1.69	−0.54	−0.43	−0.97	−0.71
TPSSh	1.20	−0.34	−0.26	−0.60	−0.60
TPSS	0.24	−0.03	−0.02	−0.05	−0.19
BHLYP	2.50	−0.81	−0.66	−1.47	−1.03
PBE0	2.06	−0.61	−0.51	−1.12	−0.94
B97−D	0.71	−0.16	−0.12	−0.28	−0.42
M06	1.90	−0.57	−0.48	−1.05	−0.84
M06−L	1.03	−0.26	−0.21	−0.47	−0.56
M06−2X	2.34	−0.71	−0.66	−1.37	−0.96
DMRG	0.00	0.00	0.00	0.00	0.00

Table S7. Spin population values in ^{4}{Fe[P](ImH)(NO)}, calculated with different functionals and DMRG

Complex	Fe	N	O	NO	The rest
BP86	3.17	−0.25	−0.22	−0.46	0.29
PBE	3.16	−0.24	−0.21	−0.46	0.29
B3LYP	3.75	−0.56	−0.50	−1.06	0.31
TPSSh	3.62	−0.48	−0.41	−0.89	0.28
TPSS	3.22	−0.27	−0.23	−0.50	0.28
BHLYP	4.14	−0.75	−0.68	−1.43	0.28
PBE0	3.90	−0.62	−0.55	−1.17	0.26
B97−D	3.40	−0.39	−0.32	−0.71	0.31
M06	3.82	−0.58	−0.52	−1.11	0.29
M06−L	3.63	−0.47	−0.44	−0.91	0.28
M06−2X	4.05	−0.70	−0.69	−1.39	0.34
DMRG	3.33	−0.28	−0.20	−0.48	0.15

Table S8. Spin population values in ^{2}{Fe[P](ImH)(NO)}, calculated with different functionals and DMRG

Complex	Fe	N	O	NO	The rest
BP86	0.53	0.29	0.17	0.46	0.01
PBE	0.54	0.28	0.17	0.45	0.01
B3LYP	0.28	0.45	0.27	0.72	0.00
TPSSh	0.35	0.41	0.25	0.66	−0.01
TPSS	0.46	0.34	0.20	0.54	0.00
BHLYP	0.10	0.58	0.31	0.88	0.01
PBE0	0.25	0.47	0.28	0.75	−0.01
B97−D	0.61	0.25	0.14	0.39	0.00
M06	0.41	0.37	0.22	0.59	0.00
M06−L	0.51	0.31	0.19	0.50	−0.01
M06−2X	0.09	0.58	0.33	0.91	0.01
DMRG	0.81	0.11	0.07	0.18	0.00

Table S9. Spin population values in $^{3}\{Fe[P](ImH)(NO)\}^{+}$, calculated with different functionals and DMRG

Complex	Fe	N	O	NO	The rest
BP86	1.42	0.09	0.03	0.12	0.47
PBE	1.43	0.08	0.03	0.11	0.46
B3LYP	2.59	−0.24	−0.28	−0.52	−0.07
TPSSh	–	–	–	–	–
TPSS	1.29	0.13	0.07	0.20	0.51
BHLYP	3.10	−0.46	−0.41	−0.87	−0.24
PBE0	2.87	−0.33	−0.37	−0.70	−0.17
B97−D	1.82	−0.08	−0.10	−0.18	0.36
M06	2.92	−0.34	−0.39	−0.73	−0.19
M06−L	1.92	−0.08	−0.13	−0.21	0.29
M06−2X	3.04	−0.43	−0.40	−0.83	−0.21
DMRG	2.02	0.04	−0.02	0.02	−0.04

Table S10. Spin population values in $^{1}\{Fe[P](ImH)(NO)\}^{+}$, calculated with different functionals and DMRG

Complex	Fe	N	O	NO	The rest
BP86	0.03	−0.01	−0.01	−0.02	−0.01
PBE	0.01	0.00	0.00	0.00	−0.01
B3LYP	0.52	−0.23	−0.21	−0.45	−0.07
TPSSh	0.25	−0.11	−0.10	−0.21	−0.03
TPSS	0.02	0.00	0.00	0.00	−0.02
BHLYP	0.98	−0.49	−0.37	−0.85	−0.13
PBE0	0.67	−0.30	−0.28	−0.58	−0.09
B97−D	0.05	−0.02	−0.02	−0.04	−0.01
M06	0.47	−0.20	−0.20	−0.40	−0.07
M06−L	0.06	−0.03	−0.03	−0.05	−0.01
M06−2X	0.92	−0.44	−0.39	−0.83	−0.09
DMRG	0.00	0.00	0.00	0.00	0.00

Table S11. Spin population values in ^{3}{Fe[P](NO_2)(NO)}, calculated with different functionals and DMRG

Complex	Fe	N	O	NO	NO_2	The rest
BP86	0.95	0.29	0.21	0.50	0.44	0.12
PBE	0.96	0.28	0.21	0.49	0.44	0.12
B3LYP	0.86	0.38	0.26	0.64	0.43	0.07
TPSSh	0.89	0.36	0.25	0.61	0.45	0.06
TPSS	0.90	0.32	0.23	0.55	0.44	0.11
BHLYP	0.65	0.52	0.31	0.82	0.47	0.06
PBE0	0.86	0.40	0.27	0.67	0.45	0.03
B97–D	1.05	0.24	0.18	0.42	0.43	0.10
M06	1.03	0.30	0.22	0.52	0.41	0.04
M06–L	1.00	0.26	0.20	0.46	0.47	0.08
M06–2X	0.79	0.50	0.32	0.82	0.37	0.03
DMRG	1.17	0.46	0.31	0.77	0.06	0.00

Table S12. Spin population values in ^{1}{Fe[P](NO_2)(NO)}, calculated with different functionals and DMRG

Complex	Fe	N	O	NO	NO_2	The rest
BP86	0.07	−0.03	−0.02	−0.05	0.00	−0.02
PBE	0.03	−0.01	0.00	−0.01	0.00	−0.01
B3LYP	0.79	−0.35	−0.30	−0.65	0.06	−0.20
TPSSh	0.45	−0.21	−0.17	−0.38	0.03	−0.09
TPSS	0.08	−0.04	−0.03	−0.07	0.00	−0.01
BHLYP	1.15	−0.52	−0.38	−0.89	0.13	−0.39
PBE0	0.94	−0.40	−0.36	−0.76	0.08	−0.26
B97–D	0.09	−0.04	−0.03	−0.06	0.00	−0.03
M06	0.80	−0.30	−0.28	−0.58	0.13	−0.34
M06–L	0.19	−0.07	−0.07	−0.14	0.01	−0.06
M06–2X	1.18	−0.48	−0.41	−0.88	0.20	−0.50
DMRG	0.00	0.00	0.00	0.00	0.00	0.00

Table S13. Spin population values in ^{3}{Fe[P](SMe)(NO)}, calculated with different functionals and DMRG

Complex	Fe	N	O	NO	SMe	The rest
BP86	0.78	0.38	0.27	0.66	0.57	−0.01
PBE	0.79	0.38	0.27	0.65	0.56	0.00
B3LYP	0.77	0.47	0.30	0.77	0.48	−0.01
TPSSh	0.78	0.45	0.30	0.75	0.49	−0.02
TPSS	0.76	0.41	0.29	0.70	0.53	0.01
BHLYP	0.63	0.55	0.31	0.86	0.49	0.02
PBE0	0.80	0.48	0.31	0.78	0.45	−0.04
B97−D	0.83	0.36	0.25	0.61	0.56	−0.01
M06	0.86	0.41	0.28	0.69	0.45	0.00
M06−L	0.90	0.35	0.26	0.61	0.49	0.00
M06−2X	0.85	0.55	0.33	0.88	0.26	0.00
DMRG	1.00	0.58	0.32	0.90	0.10	0.01

Table S14. Spin population values in ^{1}{Fe[P](SMe)(NO)}, calculated with different functionals and DMRG

Complex	Fe	N	O	NO	SMe	The rest
BP86	0.03	−0.02	−0.01	−0.03	0.00	0.00
PBE	0.04	−0.02	−0.02	−0.04	0.00	0.00
B3LYP	0.87	−0.43	−0.35	−0.78	0.00	−0.09
TPSSh	0.53	−0.30	−0.22	−0.52	0.05	−0.06
TPSS	0.12	−0.07	−0.05	−0.13	0.02	−0.02
BHLYP	1.53	−0.55	−0.41	−0.96	−0.40	−0.16
PBE0	1.02	−0.48	−0.40	−0.88	−0.02	−0.12
B97−D	0.12	−0.06	−0.04	−0.10	0.00	−0.02
M06	0.89	−0.37	−0.33	−0.70	−0.08	−0.11
M06−L	0.25	−0.13	−0.10	−0.23	0.02	−0.04
M06−2X	1.56	−0.53	−0.50	−1.03	−0.39	−0.13
DMRG	0.00	0.00	0.00	0.00	0.00	0.00

Bond distances and vibrational frequencies

Table S15. Selected bond distances[c] (in Å), Fe−NO bond angle (in degree), and NO vibrational wavenumber (in cm^{-1})

	Fe-X_{axial}[b]	mean Fe-N_{eq}[a]	Fe-NO	N-O	∠ FeNO	$\tilde{\nu}$
^{4}\{Fe[P](NO)\}	–	2.095 (2.105)	1.725 (1.728)	1.170 (1.170)	153.8 (158.4)	1770.7
^{2}\{Fe[P](NO)\}	–	2.012 (2.018)	1.695 (1.697)	1.177 (1.176)	144.7 (146.2)	1708.6
^{3}\{Fe[P](NO)\}$^{+}$	–	1.976 (1.98)	1.733 (1.75)	1.160	142.1 (146)	1766.7
^{1}\{Fe[P](NO)\}$^{+}$	–	2.000 (1.99)	1.610 (1.60)	1.153	180.0 (180)	1907.4
^{3}\{Fe[P](NO)\}$^{-}$	–	2.036	1.688	1.185	144.8	1672.7
^{1}\{Fe[P](NO)\}$^{-}$	–	2.004 (2.011)	1.773 (1.786)	1.203 (1.206)	125.0 (125)	1528.5 (1533)
^{4}\{Fe[P](ImH)(NO)\}	2.298 (2.363)	2.092 (2.091)	1.748 (1.757)	1.175 (1.174)	143.5 (144.6)	1718.4
^{2}\{Fe[P](ImH)(NO)\}	2.115 (2.201)	2.016 (2.020)	1.733 (1.731)	1.182 (1.182)	139.8 (140.7)	1674.5
^{3}\{Fe[P](ImH)(NO)\}$^{+}$	2.469	2.014	1.742	1.164	141.4	1755.9
^{1}\{Fe[P](ImH)(NO)\}$^{+}$	1.994	2.012	1.631	1.144	179.2	1945.2
^{3}\{Fe[P](NO_2)(NO)\}	2.143	2.006	1.799	1.172	134.2	1703.7
^{1}\{Fe[P](NO_2)(NO)\}	2.038 (2.021)	2.016 (1.992)	1.669 (1.675)	1.157 (1.152)	159.9 (156.4)	1849.8
^{3}\{Fe[P](SMe)(NO)\}	2.236	2.011	1.855	1.172	133.2	1715.1
^{1}\{Fe[P](SMe)(NO)\}	2.270	2.016 (2.024)	1.681 (1.709)	1.161 (1.166)	160.6 (167.8)	1826.5 (1859)

[a]Mean of all equatorial Fe-N bonds.

[b]Distance between Fe and the axial ligand atom: N in Fe[P](ImH)(NO) and Fe[P](NO_2)(NO), S in Fe[P](SMe)(NO).

[c]Numbers in brackets are from previous DFT studies: Ref. [1] for Fe[P](NO) and Fe[P](ImH)(NO), Ref. [2] for \{Fe[P](ImH)(NO)\}$^{+}$, Ref. [3] for \{Fe[P](NO)\}$^{-}$, Ref. [4] for ^{1}\{Fe[P](NO_2)(NO)\}, Ref. [5] and [6] for ^{1}\{Fe[P](SMe)(NO)\}.

Table S16. Corelation between charge, vibrational frequency, and bond length of NO

NO charge	Vibrational wavenumber (cm^{-1})	Bond length (Å)
1.0	2365.2	1.070
0.9	2317.8	1.077
0.8	2271.9	1.086
0.7	2225.6	1.094
0.6	2178.7	1.102
0.5	2131.8	1.111
0.4	2083.8	1.120
0.3	2035.7	1.129
0.2	1986.8	1.139
0.1	1938.2	1.148
0.0	1889.2	1.158
–0.1	1837.9	1.169
–0.2	1787.3	1.179
–0.3	1736.7	1.190
–0.4	1685.0	1.202
–0.5	1635.0	1.213
–0.6	1584.2	1.225
–0.7	1532.0	1.238
–0.8	1480.9	1.251
–0.9	1429.2	1.265
–1.0	1375.0	1.280

Cartesian coordinates

$^{4}\{Fe[P](NO)\}$

Fe	-0.0170671	-0.3736166	0.0000000
N	1.4126474	0.1250079	1.4441583
N	1.4126474	0.1250079	-1.4441583
N	-1.4776436	0.0402883	1.4470312
N	-1.4776436	0.0402883	-1.4470312
C	3.4023362	0.1878267	0.0000000
C	2.7741793	0.1772062	1.2444109
C	2.7741793	0.1772062	-1.2444109
C	3.4508823	0.2436462	2.5203225
C	3.4508823	0.2436462	-2.5203225
C	2.4833086	0.2275808	3.4863182
C	2.4833086	0.2275808	-3.4863182
C	1.2094568	0.1498270	2.8069460
C	1.2094568	0.1498270	-2.8069460
C	-0.0343704	0.1230901	3.4351421
C	-0.0343704	0.1230901	-3.4351421
C	-1.2792596	0.0885756	2.8077131
C	-1.2792596	0.0885756	-2.8077131
C	-2.5556465	0.1238955	3.4869956
C	-2.5556465	0.1238955	-3.4869956
C	-3.5213405	0.0988891	2.5194977
C	-3.5213405	0.0988891	-2.5194977
C	-2.8391208	0.0482855	1.2450652
C	-2.8391208	0.0482855	-1.2450652
C	-3.4666501	0.0351257	0.0000000
H	4.4918543	0.2314777	0.0000000
H	4.5279484	0.3070930	2.6496797
H	4.5279484	0.3070930	-2.6496797
H	2.6100886	0.2738262	4.5645734
H	2.6100886	0.2738262	-4.5645734
H	-0.0340517	0.1548080	4.5251553
H	-0.0340517	0.1548080	-4.5251553
H	-2.6855862	0.1748306	4.5646712
H	-2.6855862	0.1748306	-4.5646712
H	-4.6002882	0.1240243	2.6464897
H	-4.6002882	0.1240243	-2.6464897
H	-4.5571315	0.0437112	0.0000000
N	0.2854928	-2.0716173	0.0000000
O	0.9787572	-3.0146561	0.0000000

^{2}{Fe[P](NO)}

Fe	-0.0083886	-0.1652002	0.0000000
N	1.3755462	0.1453528	1.4072805
N	1.3755462	0.1453528	-1.4072805
N	-1.4428690	0.0564060	1.4136646
N	-1.4428690	0.0564060	-1.4136646
C	3.3897002	0.1287367	0.0000000
C	2.7460560	0.1489274	1.2253437
C	2.7460560	0.1489274	-1.2253437
C	3.4237741	0.2060055	2.4954072
C	3.4237741	0.2060055	-2.4954072
C	2.4571109	0.2353737	3.4579941
C	2.4571109	0.2353737	-3.4579941
C	1.1877942	0.1855284	2.7781490
C	1.1877942	0.1855284	-2.7781490
C	-0.0370307	0.1717690	3.4224902
C	-0.0370307	0.1717690	-3.4224902
C	-1.2630990	0.1151311	2.7809634
C	-1.2630990	0.1151311	-2.7809634
C	-2.5345409	0.1042470	3.4611285
C	-2.5345409	0.1042470	-3.4611285
C	-3.4954531	0.0300407	2.4954486
C	-3.4954531	0.0300407	-2.4954486
C	-2.8096715	0.0021115	1.2268399
C	-2.8096715	0.0021115	-1.2268399
C	-3.4506923	-0.0449059	0.0000000
H	4.4792727	0.1290754	0.0000000
H	4.5030828	0.2313696	2.6163890
H	4.5030828	0.2313696	-2.6163890
H	2.5751044	0.2870407	4.5367216
H	2.5751044	0.2870407	-4.5367216
H	-0.0354887	0.2086628	4.5115556
H	-0.0354887	0.2086628	-4.5115556
H	-2.6578292	0.1528950	4.5394394
H	-2.6578292	0.1528950	-4.5394394
H	-4.5753037	0.0070329	2.6128438
H	-4.5753037	0.0070329	-2.6128438
H	-4.5395271	-0.0896200	0.0000000
N	0.2406995	-1.8421191	0.0000000
O	1.0545699	-2.6917551	0.0000000

$^{3}\{Fe[P](NO)\}^{+}$

Fe	-0.0110768	-0.1649008	0.0000000
N	1.3633619	0.1225402	1.3779954
N	1.3633619	0.1225402	-1.3779954
N	-1.4181085	0.0510009	1.3831328
N	-1.4181085	0.0510009	-1.3831328
C	3.3366424	-0.3405706	0.0000000
C	2.7125270	-0.1414869	1.2258755
C	2.7125270	-0.1414869	-1.2258755
C	3.3895590	0.0220457	2.4833239
C	3.3895590	0.0220457	-2.4833239
C	2.4518678	0.4376158	3.3894446
C	2.4518678	0.4376158	-3.3894446
C	1.1864228	0.4611834	2.7075590
C	1.1864228	0.4611834	-2.7075590
C	-0.0457709	0.6456743	3.3230949
C	-0.0457709	0.6456743	-3.3230949
C	-1.2668019	0.4025713	2.7110786
C	-1.2668019	0.4025713	-2.7110786
C	-2.5321295	0.3051289	3.3923458
C	-2.5321295	0.3051289	-3.3923458
C	-3.4387559	-0.1638880	2.4839571
C	-3.4387559	-0.1638880	-2.4839571
C	-2.7457990	-0.2912473	1.2273533
C	-2.7457990	-0.2912473	-1.2273533
C	-3.3540813	-0.5304310	0.0000000
H	4.4106213	-0.5228306	0.0000000
H	4.4550572	-0.1221699	2.6354321
H	4.4550572	-0.1221699	-2.6354321
H	2.5926225	0.6882192	4.4366347
H	2.5926225	0.6882192	-4.4366347
H	-0.0486778	0.8879570	4.3852247
H	-0.0486778	0.8879570	-4.3852247
H	-2.6899450	0.5456226	4.4394700
H	-2.6899450	0.5456226	-4.4394700
H	-4.4947747	-0.3697530	2.6313752
H	-4.4947747	-0.3697530	-2.6313752
H	-4.4156151	-0.7758774	0.0000000
N	0.1492037	-1.8907073	0.0000000
O	0.9429960	-2.7367107	0.0000000

$^{1}\{Fe[P](NO)\}^{+}$

Fe	0.0077690	-0.2041596	0.0000000
N	1.3907964	0.1792822	1.3924020
N	1.3907964	0.1792822	-1.3924020
N	-1.4003891	0.0752131	1.3924773
N	-1.4003891	0.0752131	-1.3924773
C	3.4165298	0.1431510	0.0000000
C	2.7705148	0.1661114	1.2194928
C	2.7705148	0.1661114	-1.2194928
C	3.4440431	0.2353719	2.4858407
C	3.4440431	0.2353719	-2.4858407
C	2.4778396	0.2811330	3.4467692
C	2.4778396	0.2811330	-3.4467692
C	1.2122306	0.2260785	2.7702375
C	1.2122306	0.2260785	-2.7702375
C	-0.0082528	0.2199453	3.4143454
C	-0.0082528	0.2199453	-3.4143454
C	-1.2259075	0.1365064	2.7701594
C	-1.2259075	0.1365064	-2.7701594
C	-2.4921122	0.0963065	3.4466507
C	-2.4921122	0.0963065	-3.4466507
C	-3.4518978	-0.0250312	2.4858350
C	-3.4518978	-0.0250312	-2.4858350
C	-2.7749703	-0.0440362	1.2195455
C	-2.7749703	-0.0440362	-1.2195455
C	-3.4171530	-0.1177002	0.0000000
H	4.5058178	0.1395932	0.0000000
H	4.5228830	0.2636629	2.6065523
H	4.5228830	0.2636629	-2.6065523
H	2.5949822	0.3522525	4.5240270
H	2.5949822	0.3522525	-4.5240270
H	-0.0101123	0.2692642	4.5025179
H	-0.0101123	0.2692642	-4.5025179
H	-2.6142215	0.1595781	4.5238461
H	-2.6142215	0.1595781	-4.5238461
H	-4.5297312	-0.0796369	2.6064598
H	-4.5297312	-0.0796369	-2.6064598
H	-4.5028712	-0.2057899	0.0000000
N	0.0673926	-1.8133685	0.0000000
O	0.1111251	-2.9657296	0.0000000

$^{3}\{Fe[P](NO)\}^{-}$

Fe	-0.0092925	-0.1836848	0.0000000
N	1.3885460	0.1408372	1.4074774
N	1.3885460	0.1408372	-1.4074774
N	-1.4536508	0.0650781	1.4144183
N	-1.4536508	0.0650781	-1.4144183
C	3.4067119	0.0265730	0.0000000
C	2.7557519	0.0949085	1.2293990
C	2.7557519	0.0949085	-1.2293990
C	3.4267518	0.1756504	2.5002255
C	3.4267518	0.1756504	-2.5002255
C	2.4515967	0.2767452	3.4630151
C	2.4515967	0.2767452	-3.4630151
C	1.1913045	0.2402627	2.7881610
C	1.1913045	0.2402627	-2.7881610
C	-0.0383869	0.2638436	3.4220068
C	-0.0383869	0.2638436	-3.4220068
C	-1.2683122	0.1728753	2.7912825
C	-1.2683122	0.1728753	-2.7912825
C	-2.5296961	0.1415257	3.4665883
C	-2.5296961	0.1415257	-3.4665883
C	-3.4962305	-0.0029481	2.5005509
C	-3.4962305	-0.0029481	-2.5005509
C	-2.8156299	-0.0456163	1.2314403
C	-2.8156299	-0.0456163	-1.2314403
C	-3.4597989	-0.1453540	0.0000000
H	4.4957440	-0.0076868	0.0000000
H	4.5067932	0.1730813	2.6255397
H	4.5067932	0.1730813	-2.6255397
H	2.5685849	0.3631511	4.5409224
H	2.5685849	0.3631511	-4.5409224
H	-0.0374628	0.3388483	4.5108441
H	-0.0374628	0.3388483	-4.5108441
H	-2.6536973	0.2201273	4.5444024
H	-2.6536973	0.2201273	-4.5444024
H	-4.5750966	-0.0622444	2.6227370
H	-4.5750966	-0.0622444	-2.6227370
H	-4.5463593	-0.2312008	0.0000000
N	0.2287835	-1.8544341	0.0000000
O	1.0418796	-2.7164642	0.0000000

$^{1}\{Fe[P](NO)\}^{-}$

Fe	-0.0027672	-0.1151084	0.0000000
N	1.3795523	0.1311398	1.4035002
N	1.3795523	0.1311398	-1.4035002
N	-1.4419705	0.0310624	1.4129667
N	-1.4419705	0.0310624	-1.4129667
C	3.3968308	0.1177804	0.0000000
C	2.7537951	0.1360172	1.2261153
C	2.7537951	0.1360172	-1.2261153
C	3.4340489	0.1957219	2.4978335
C	3.4340489	0.1957219	-2.4978335
C	2.4624052	0.2357534	3.4586567
C	2.4624052	0.2357534	-3.4586567
C	1.1944687	0.1872337	2.7770299
C	1.1944687	0.1872337	-2.7770299
C	-0.0343211	0.1840492	3.4204104
C	-0.0343211	0.1840492	-3.4204104
C	-1.2622338	0.1203621	2.7853124
C	-1.2622338	0.1203621	-2.7853124
C	-2.5328485	0.1189809	3.4650777
C	-2.5328485	0.1189809	-3.4650777
C	-3.4957040	0.0207874	2.4989879
C	-3.4957040	0.0207874	-2.4989879
C	-2.8082022	-0.0257851	1.2289422
C	-2.8082022	-0.0257851	-1.2289422
C	-3.4507172	-0.0870533	0.0000000
H	4.4877310	0.1192501	0.0000000
H	4.5143770	0.2163972	2.6194845
H	4.5143770	0.2163972	-2.6194845
H	2.5779204	0.2889767	4.5390784
H	2.5779204	0.2889767	-4.5390784
H	-0.0314294	0.2373834	4.5104593
H	-0.0314294	0.2373834	-4.5104593
H	-2.6563787	0.1814836	4.5440737
H	-2.6563787	0.1814836	-4.5440737
H	-4.5768551	0.0005161	2.6162629
H	-4.5768551	0.0005161	-2.6162629
H	-4.5404616	-0.1351286	0.0000000
N	0.0711468	-1.8863091	0.0000000
O	1.0849887	-2.5335906	0.0000000

^{4}{Fe[P](ImH)(NO)}

Fe	0.0428609	0.7992381	0.0000000
N	-1.4586538	0.6942001	-1.4665996
N	-1.4586538	0.6942001	1.4665996
N	1.4854012	0.4884965	1.4675869
N	1.4854012	0.4884965	-1.4675869
N	-0.1041434	-1.4943501	0.0000000
N	-0.9637398	-3.5202776	0.0000000
C	-2.8106725	0.7709526	1.2565051
C	-2.8106725	0.7709526	-1.2565051
C	1.2628660	0.4525829	2.8198216
C	1.2628660	0.4525829	-2.8198216
C	-1.2462471	0.6140275	2.8184098
C	-1.2462471	0.6140275	-2.8184098
C	-2.5254818	0.6506883	3.5022678
C	-2.5254818	0.6506883	-3.5022678
C	-3.4919159	0.7490719	2.5379279
C	-3.4919159	0.7490719	-2.5379279
C	3.5107700	0.3413310	2.5389376
C	3.5107700	0.3413310	-2.5389376
C	-3.4298292	0.8190550	0.0000000
C	3.4579322	0.4088035	0.0000000
C	0.0064501	0.5060608	3.4352825
C	0.0064501	0.5060608	-3.4352825
C	2.8382017	0.4258470	1.2565532
C	2.8382017	0.4258470	-1.2565532
C	2.5387587	0.3537125	3.5026843
C	2.5387587	0.3537125	-3.5026843
C	-1.2333672	-2.1840543	0.0000000
C	0.4117132	-3.6813566	0.0000000
C	0.9286766	-2.4075050	0.0000000
H	-4.5193473	0.8776210	0.0000000
H	-4.5691771	0.8001297	2.6743009
H	-4.5691771	0.8001297	-2.6743009
H	-2.6595593	0.6048680	4.5799825
H	-2.6595593	0.6048680	-4.5799825
H	0.0035706	0.4606122	4.5253522
H	0.0035706	0.4606122	-4.5253522
H	2.6667965	0.3028787	4.5808152
H	2.6667965	0.3028787	-4.5808152
H	4.5868662	0.2738923	2.6761100
H	4.5868662	0.2738923	-2.6761100
H	4.5475166	0.3561256	0.0000000
H	-2.2328171	-1.7630067	0.0000000
H	-1.6503469	-4.2655353	0.0000000
H	0.8771013	-4.6592856	0.0000000
H	1.9635929	-2.0857412	0.0000000
N	0.3800993	2.5148074	0.0000000
O	1.2481512	3.3067579	0.0000000

^{2}{Fe[P](ImH)(NO)}

Fe	0.0329866	0.6522633	0.0000000
N	-1.4111905	0.6478977	-1.4231003
N	-1.4111905	0.6478977	1.4231003
N	1.4379867	0.4779182	1.4172530
N	1.4379867	0.4779182	-1.4172530
N	-0.0942523	-1.4592115	0.0000000
N	-0.9318278	-3.4905720	0.0000000
C	-2.7694707	0.7677967	1.2330974
C	-2.7694707	0.7677967	-1.2330974
C	1.2380859	0.4207111	2.7794591
C	1.2380859	0.4207111	-2.7794591
C	-1.2235426	0.5614944	2.7840789
C	-1.2235426	0.5614944	-2.7840789
C	-2.4945538	0.6275429	3.4688184
C	-2.4945538	0.6275429	-3.4688184
C	-3.4538540	0.7612928	2.5068199
C	-3.4538540	0.7612928	-2.5068199
C	3.4786062	0.3919531	2.5059216
C	3.4786062	0.3919531	-2.5059216
C	-3.4055402	0.8396585	0.0000000
C	3.4439596	0.4749272	0.0000000
C	0.0058613	0.4452609	3.4175325
C	0.0058613	0.4452609	-3.4175325
C	2.8027553	0.4647183	1.2312177
C	2.8027553	0.4647183	-1.2312177
C	2.5081750	0.3563841	3.4649303
C	2.5081750	0.3563841	-3.4649303
C	-1.2189958	-2.1601892	0.0000000
C	0.4449141	-3.6372379	0.0000000
C	0.9504946	-2.3600815	0.0000000
H	-4.4916244	0.9337902	0.0000000
H	-4.5302219	0.8458084	2.6305179
H	-4.5302219	0.8458084	-2.6305179
H	-2.6188486	0.5831261	4.5474539
H	-2.6188486	0.5831261	-4.5474539
H	0.0059125	0.3909319	4.5062675
H	0.0059125	0.3909319	-4.5062675
H	2.6240809	0.3008328	4.5438976
H	2.6240809	0.3008328	-4.5438976
H	4.5575297	0.3665782	2.6318871
H	4.5575297	0.3665782	-2.6318871
H	4.5337981	0.4638594	0.0000000
H	-2.2217815	-1.7482277	0.0000000
H	-1.6109020	-4.2426079	0.0000000
H	0.9213085	-4.6095986	0.0000000
H	1.9810603	-2.0262415	0.0000000
N	0.2427238	2.3722675	0.0000000
O	1.1090553	3.1767063	0.0000000

$^{3}\{Fe[P](ImH)(NO)\}^{+}$

Fe	0.0342544	0.7589771	0.0000000
N	-1.4020792	0.7167350	-1.4239725
N	-1.4020792	0.7167350	1.4239725
N	1.4266425	0.5217509	1.4228887
N	1.4266425	0.5217509	-1.4228887
N	-0.1174837	-1.7054567	0.0000000
N	-0.9388302	-3.7481370	0.0000000
C	-2.7596130	0.5746917	1.2377099
C	-2.7596130	0.5746917	-1.2377099
C	1.2573600	0.7400967	2.7733700
C	1.2573600	0.7400967	-2.7733700
C	-1.2103538	0.9061132	2.7756812
C	-1.2103538	0.9061132	-2.7756812
C	-2.4824176	0.8933841	3.4568462
C	-2.4824176	0.8933841	-3.4568462
C	-3.4382621	0.6616263	2.5091581
C	-3.4382621	0.6616263	-2.5091581
C	3.4394129	0.2235194	2.5085328
C	3.4394129	0.2235194	-2.5085328
C	-3.3894319	0.4798164	0.0000000
C	3.3749593	0.0555470	0.0000000
C	0.0324370	0.9617278	3.3978689
C	0.0324370	0.9617278	-3.3978689
C	2.7596304	0.2215285	1.2368056
C	2.7596304	0.2215285	-1.2368056
C	2.5161745	0.5687041	3.4550903
C	2.5161745	0.5687041	-3.4550903
C	-1.2351642	-2.4196416	0.0000000
C	0.4356713	-3.8849902	0.0000000
C	0.9312895	-2.6026698	0.0000000
H	-4.4768682	0.4045542	0.0000000
H	-4.5126691	0.5768610	2.6435218
H	-4.5126691	0.5768610	-2.6435218
H	-2.6128173	1.0219793	4.5272795
H	-2.6128173	1.0219793	-4.5272795
H	0.0435393	1.0966217	4.4792285
H	0.0435393	1.0966217	-4.4792285
H	2.6586240	0.6777990	4.5261059
H	2.6586240	0.6777990	-4.5261059
H	4.4941248	0.0038939	2.6451601
H	4.4941248	0.0038939	-2.6451601
H	4.4456270	-0.1477713	0.0000000
H	-2.2453361	-2.0270137	0.0000000
H	-1.6114166	-4.5076680	0.0000000
H	0.9210178	-4.8530460	0.0000000
H	1.9636025	-2.2754430	0.0000000
N	0.3129244	2.4784826	0.0000000
O	1.1757181	3.2603952	0.0000000

$^{1}\{Fe[P](ImH)(NO)\}^{+}$

Fe	0.0393760	0.6332443	0.0000000
N	-1.3907670	0.6187509	-1.4155744
N	-1.3907670	0.6187509	1.4155744
N	1.4549032	0.4337990	1.4163184
N	1.4549032	0.4337990	-1.4163184
N	-0.0805520	-1.3567984	0.0000000
N	-0.9049646	-3.3796281	0.0000000
C	-2.7394445	0.8464201	1.2283207
C	-2.7394445	0.8464201	-1.2283207
C	1.2491232	0.3012496	2.7731889
C	1.2491232	0.3012496	-2.7731889
C	-1.2032710	0.4609810	2.7731456
C	-1.2032710	0.4609810	-2.7731456
C	-2.4695562	0.5716470	3.4525124
C	-2.4695562	0.5716470	-3.4525124
C	-3.4151966	0.8265522	2.5012201
C	-3.4151966	0.8265522	-2.5012201
C	3.4893939	0.3787377	2.5008197
C	3.4893939	0.3787377	-2.5008197
C	-3.3691898	0.9863585	0.0000000
C	3.4631207	0.5453351	0.0000000
C	0.0163656	0.2791480	3.4062014
C	0.0163656	0.2791480	-3.4062014
C	2.8209972	0.4859829	1.2281146
C	2.8209972	0.4859829	-1.2281146
C	2.5195348	0.2472227	3.4523260
C	2.5195348	0.2472227	-3.4523260
C	-1.2104389	-2.0621164	0.0000000
C	0.4707047	-3.5206112	0.0000000
C	0.9771589	-2.2470941	0.0000000
H	-4.4438092	1.1632061	0.0000000
H	-4.4827151	0.9775996	2.6323869
H	-4.4827151	0.9775996	-2.6323869
H	-2.5998920	0.4780904	4.5266636
H	-2.5998920	0.4780904	-4.5266636
H	0.0089819	0.1671866	4.4894882
H	0.0089819	0.1671866	-4.4894882
H	2.6370932	0.1353461	4.5261646
H	2.6370932	0.1353461	-4.5261646
H	4.5675581	0.3891819	2.6310151
H	4.5675581	0.3891819	-2.6310151
H	4.5515477	0.5817084	0.0000000
H	-2.2153771	-1.6592031	0.0000000
H	-1.5805605	-4.1368385	0.0000000
H	0.9552748	-4.4885925	0.0000000
H	2.0065563	-1.9150115	0.0000000
N	0.1581191	2.2600068	0.0000000
O	0.2568165	3.4002428	0.0000000

$^3\{Fe[P](NO_2)(NO)\}$

Fe	0.0301789	-0.1472911	0.0000000
N	1.4056087	-0.0354698	1.4125619
N	1.4056087	-0.0354698	-1.4125619
N	-1.4146100	-0.1306883	1.4362046
N	-1.4146100	-0.1306883	-1.4362046
C	3.4139979	-0.0844919	0.0000000
C	2.7727690	-0.0550191	1.2270317
C	2.7727690	-0.0550191	-1.2270317
C	3.4500264	0.0047246	2.4971936
C	3.4500264	0.0047246	-2.4971936
C	2.4853235	0.0690349	3.4604168
C	2.4853235	0.0690349	-3.4604168
C	1.2138615	0.0339827	2.7862601
C	1.2138615	0.0339827	-2.7862601
C	-0.0084078	0.0400044	3.4326060
C	-0.0084078	0.0400044	-3.4326060
C	-1.2391966	-0.0484799	2.7955816
C	-1.2391966	-0.0484799	-2.7955816
C	-2.5134755	-0.0878516	3.4698283
C	-2.5134755	-0.0878516	-3.4698283
C	-3.4655770	-0.2055563	2.4979256
C	-3.4655770	-0.2055563	-2.4979256
C	-2.7711853	-0.2235302	1.2324946
C	-2.7711853	-0.2235302	-1.2324946
C	-3.4038454	-0.2868913	0.0000000
H	4.5033814	-0.0954723	0.0000000
H	4.5295441	0.0103185	2.6182843
H	4.5295441	0.0103185	-2.6182843
H	2.6068907	0.1320229	4.5380054
H	2.6068907	0.1320229	-4.5380054
H	-0.0029682	0.0994338	4.5205024
H	-0.0029682	0.0994338	-4.5205024
H	-2.6455632	-0.0325846	4.5465857
H	-2.6455632	-0.0325846	-4.5465857
H	-4.5445758	-0.2640701	2.6096938
H	-4.5445758	-0.2640701	-2.6096938
H	-4.4914308	-0.3554647	0.0000000
N	0.0925703	-1.9454238	0.0000000
O	0.9606260	-2.7332157	0.0000000
N	-0.2584478	1.9760757	0.0000000
O	-1.3730013	2.4741829	0.0000000
O	0.8090416	2.5854599	0.0000000

$^{1}\{Fe[P](NO_2)(NO)\}$

Fe	-0.0283295	-0.1945972	0.0000000
N	1.3899354	-0.0208308	1.4335855
N	1.3899354	-0.0208308	-1.4335855
N	-1.4444433	-0.1484196	1.4234701
N	-1.4444433	-0.1484196	-1.4234701
C	3.3879917	-0.0716402	0.0000000
C	2.7512775	-0.0375750	1.2308548
C	2.7512775	-0.0375750	-1.2308548
C	3.4335763	0.0337756	2.5001356
C	3.4335763	0.0337756	-2.5001356
C	2.4719373	0.1028636	3.4662334
C	2.4719373	0.1028636	-3.4662334
C	1.1967795	0.0545446	2.7930834
C	1.1967795	0.0545446	-2.7930834
C	-0.0350021	0.0619277	3.4289768
C	-0.0350021	0.0619277	-3.4289768
C	-1.2576741	-0.0437859	2.7847974
C	-1.2576741	-0.0437859	-2.7847974
C	-2.5317581	-0.0798447	3.4608043
C	-2.5317581	-0.0798447	-3.4608043
C	-3.4880232	-0.2162923	2.4973943
C	-3.4880232	-0.2162923	-2.4973943
C	-2.8033422	-0.2490600	1.2279655
C	-2.8033422	-0.2490600	-1.2279655
C	-3.4401238	-0.3255188	0.0000000
H	4.4773088	-0.0820253	0.0000000
H	4.5134558	0.0463432	2.6166758
H	4.5134558	0.0463432	-2.6166758
H	2.5961026	0.1791703	4.5426414
H	2.5961026	0.1791703	-4.5426414
H	-0.0421940	0.1368530	4.5157323
H	-0.0421940	0.1368530	-4.5157323
H	-2.6583514	-0.0063340	4.5371728
H	-2.6583514	-0.0063340	-4.5371728
H	-4.5662490	-0.2754067	2.6153575
H	-4.5662490	-0.2754067	-2.6153575
H	-4.5267888	-0.4023490	0.0000000
N	0.2862489	-1.8335426	0.0000000
O	0.8817905	-2.8250788	0.0000000
N	-0.0143352	1.8435307	0.0000000
O	-1.1251439	2.3674682	0.0000000
O	1.0493265	2.4479066	0.0000000

^{3}{Fe[P](SMe)(NO)}

Fe	0.0109041	-0.2320991	-0.1642231
N	1.3863184	-0.0937411	1.2710836
N	1.4316652	-0.1612089	-1.5661010
N	-1.4351972	-0.3317102	1.2516104
N	-1.3948919	-0.3461652	-1.6187148
C	3.3856856	0.2060633	-0.1256809
C	2.7371284	0.1165439	1.0972091
C	2.7771376	0.0509358	-1.3611525
C	3.3994692	0.1734695	2.3778875
C	3.4766497	0.0610878	-2.6238720
C	2.4417173	-0.0264360	3.3299668
C	2.5430387	-0.1539913	-3.5951236
C	1.1870194	-0.1869456	2.6364885
C	1.2672259	-0.2784486	-2.9313151
C	-0.0372245	-0.3553167	3.2646367
C	0.0562527	-0.4239822	-3.5906602
C	-1.2636554	-0.3954254	2.6143370
C	-1.1871172	-0.4272195	-2.9744601
C	-2.5473611	-0.4523228	3.2733875
C	-2.4530673	-0.4795106	-3.6688911
C	-3.4954836	-0.4082835	2.2916179
C	-3.4273298	-0.4185694	-2.7156308
C	-2.7933914	-0.3419140	1.0306642
C	-2.7591917	-0.3453368	-1.4359589
C	-3.4114880	-0.3307281	-0.2119946
H	4.4625914	0.3718380	-0.1123376
H	4.4655332	0.3332600	2.5129727
H	4.5470541	0.2105815	-2.7338529
H	2.5564100	-0.0592194	4.4097749
H	2.6850996	-0.2133174	-4.6705424
H	-0.0402834	-0.4124146	4.3527833
H	0.0814987	-0.4962912	-4.6776455
H	-2.6876262	-0.5095810	4.3491509
H	-2.5633075	-0.5470082	-4.7475442
H	-4.5769450	-0.4257000	2.3943339
H	-4.5056453	-0.4302382	-2.8477327
H	-4.5011615	-0.3420690	-0.2270368
N	0.1321587	-2.0821582	-0.1158554
O	1.0371628	-2.8258523	-0.0974639
S	-0.3114745	1.9780175	-0.2709699
C	-0.1831158	2.6504727	1.4020927
H	-0.3231648	3.7374314	1.3420279
H	-0.9660641	2.2216055	2.0422534
H	0.7964667	2.4218977	1.8404810

^{1}\{Fe[P](SMe)(NO)\}

Fe	0.0684675	-0.3432474	-0.2027203
N	1.4648124	-0.1164860	1.2419740
N	1.4837385	-0.1423271	-1.6136028
N	-1.3715954	-0.3636701	1.2227482
N	-1.3361009	-0.3743276	-1.6360819
C	3.4339262	0.2707934	-0.1720228
C	2.8024386	0.1373414	1.0566342
C	2.8187631	0.1238317	-1.4049088
C	3.4709681	0.1912238	2.3351753
C	3.5092049	0.1953343	-2.6716789
C	2.5263232	-0.0606599	3.2887614
C	2.5810460	-0.0387641	-3.6433400
C	1.2715340	-0.2437634	2.5987805
C	1.3151371	-0.2335817	-2.9751693
C	0.0486672	-0.4424506	3.2250038
C	0.1027970	-0.4091728	-3.6251910
C	-1.1816329	-0.4606114	2.5817733
C	-1.1326128	-0.4338032	-2.9948918
C	-2.4607994	-0.5087596	3.2509173
C	-2.4021384	-0.4570938	-3.6823562
C	-3.4186976	-0.4272670	2.2812362
C	-3.3722402	-0.3912221	-2.7251885
C	-2.7297929	-0.3532480	1.0142234
C	-2.7008397	-0.3466598	-1.4477233
C	-3.3529407	-0.3261045	-0.2248055
H	4.5040271	0.4757429	-0.1664652
H	4.5311645	0.3874757	2.4677061
H	4.5707064	0.3978534	-2.7827939
H	2.6479779	-0.1056019	4.3673270
H	2.7182093	-0.0623115	-4.7207563
H	0.0488694	-0.5263744	4.3112735
H	0.1157976	-0.4634716	-4.7132348
H	-2.5906897	-0.5783692	4.3272222
H	-2.5165500	-0.5013689	-4.7617704
H	-4.4989992	-0.4245559	2.3955758
H	-4.4508924	-0.3770674	-2.8533870
H	-4.4424175	-0.3164285	-0.2382983
N	0.1940658	-2.0021807	0.0396041
O	0.2325200	-3.0306843	0.5770663
S	-0.1715658	1.9131663	-0.2486018
C	-0.0334973	2.5721578	1.4330124
H	-0.2171009	3.6525248	1.3481046
H	-0.7900986	2.1405808	2.1001442
H	0.9663242	2.4143439	1.8552279

References

[1] M. Radoń, E. Broclawik, K. Pierloot, *J. Phys. Chem. B* **2010**, *114*, 1518–1528.

[2] A. Abdurahman, T. Renger, *J. Phys. Chem. A* **2009**, *113*, 9202–9206.

[3] L. E. Goodrich, S. Roy, E. E. Alp, J. Zhao, M. Y. Hu, N. Lehnert, *Inorg. Chem.* **2013**, *52*, 7766–7780.

[4] I. V. Novozhilova, P. Coppens, J. Lee, G. B. Richter-Addo, K. A. Bagley, *J. Am. Chem. Soc.* **2006**, *128*, 2093–2104.

[5] T. Wondimagegn, A. Ghosh, *J. Am. Chem. Soc.* **2001**, *123*, 5680–5683.

[6] D. P. Linder, K. R. Rodgers, *JBIC J. Biol. Inorg. Chem.* **2007**, *12*, 721–731.
